# T-Cell Phenotypes and Systemic Cytokine Profiles of People Living with HIV Admitted to Hospital with COVID-19

**DOI:** 10.3390/microorganisms12112149

**Published:** 2024-10-25

**Authors:** Mieke A. van der Mescht, Helen C. Steel, Zelda de Beer, Andries Masenge, Fareed Abdullah, Veronica Ueckermann, Ronald Anderson, Theresa M. Rossouw

**Affiliations:** 1 Department of Immunology, School of Medicine, Faculty of Health Sciences, University of Pretoria, Pretoria 0001, South Africa; u14033692@tuks.co.za (M.A.v.d.M.); helen.steel@up.ac.za (H.C.S.); zeldavdwalt22@gmail.com (Z.d.B.); ronald.anderson@up.ac.za (R.A.); 2 Tshwane District Hospital, Pretoria 0084, South Africa; 3 Department of Statistics, Faculty of Natural and Agricultural Sciences, University of Pretoria, Pretoria 0001, South Africa; andries.masenge@up.ac.za; 4 Division for Infectious Diseases, Department of Internal Medicine, Steve Biko Academic Hospital, University of Pretoria, Pretoria 0001, South Africa; fareed.abdullah@mrc.ac.za (F.A.); veronica.ueckermann@up.ac.za (V.U.); 5 Office of AIDS and TB Research, South African Medical Research Council, Pretoria 0001, South Africa; 6 Department of Public Health Medicine, Faculty of Health Sciences, University of Pretoria, Pretoria 0001, South Africa

**Keywords:** SARS-CoV-2, COVID-19, HIV, T-cells, cytokines

## Abstract

Whether SARS-CoV-2 infection leads to a higher mortality and morbidity in people living with HIV (PLWH) in Africa remains inconclusive. In this study, we explored the differences in the T-cell phenotypes between people with and without HIV on the day of admission (V1) and ±7 days later (V2), as well as their cytokine/chemokine profiles on V1. Patients admitted with COVID-19 were recruited between May 2020 and December 2021 from the Steve Biko Academic and Tshwane District Hospitals in Pretoria, South Africa. Of 174 patients, 37 (21%) were PLWH. T-cell profiles were determined by flow cytometry, and cytokine levels were determined using a multiplex suspension bead array. PLWH were significantly younger than those without HIV, and were more likely to be female. In an adjusted analysis, PLWH had higher percentages of CD4+ central memory (CM) programmed cell death protein 1 (PD-1)+, CD8+ effector memory (EM)2, and CD8+ EM4 CD57+ cells, as well as higher concentrations of interleukin (IL)-35 at admission. PLWH with CD4+ T-cell counts of >200 cells/mm^3^ had altered CD4+ and CD8+ T-cell profiles, lower levels of systemic inflammation measured by plasma ferritin and PCT levels, and less severe disease. PLWH with CD4+ T-cell counts of <200 cells/mm^3^ on admission had higher concentrations of IL-6 and lower levels of IL-29. At V2, the percentages of CD4+ CM PD-1+ T-cells and CD8+ EM4 T-cells co-expressing CD57 and PD-1 remained higher in PLWH, while all other CD8+ EM populations were lower. Fewer CD8+ EM T-cells after ±7 days of admission may be indicative of mechanisms inhibiting EM T-cell survival, as indicated by the higher expression of IL-35 and the T-cell maturation arrest observed in PLWH. This profile was not observed in PLWH with severe immunodeficiency, highlighting the need for differentiated care in the broader PLWH population.

## 1. Introduction

South Africa, with an estimated HIV prevalence of 17.8% among adults aged 15–49 years, hosts approximately 7.6 million people living with HIV (PLWH), constituting the largest population globally [1,2]. PLWH exhibit low CD4+ and high CD8+ T-cell counts, resulting in a low CD4/CD8 ratio [3]. Their T-cells are functionally impaired, as repeated antigen stimulation leads to a loss of proliferative capacity and eventual replicative senescence [4]. In the context of COVID-19, this may lead to negative outcomes, such as an increased susceptibility to SARS-CoV-2 (as is the case with other RNA viruses like influenza), due to ongoing immune exhaustion and senescence resulting from chronic immune activation [4,5]. COVID-19 mortality has been linked to a diminished ability to produce interferon-gamma (IFN-γ) and reduced early-stage activation of CD4+ and CD8+ T-cells [6,7]. SARS-CoV-2 upregulates inhibitory immune checkpoints, causing T-cell exhaustion in early and mid-infection stages [7]. Given the limited information on the interaction between HIV- and COVID-19-related T-cell dysfunction, this study aims to elucidate this relationship [8].

The second potential adverse outcome is hyperinflammation, characterized by high levels of C-reactive protein (CRP), interleukin (IL)-6, and tumor necrosis factor alpha (TNF-α), which can increase the risk of PLWH developing acute respiratory distress syndrome (ARDS) [3,6,7]. This risk potentially extends to PLWH with immune reconstitution and viral suppression, since systemic immune activation and inflammation persist during antiretroviral therapy (ART) [8,9].

Various cytokine signatures are associated with SARS-CoV-2 infection and severe COVID-19. Apart from the most commonly studied inflammatory markers, namely IL-6 and CRP, other immune markers of note include the IL-1 family, T-helper (Th)1-related, Th2-related, and Th17-related cytokines, interferon-**γ**-related cytokines, and growth factors [10]. Robust associations have been reported between elevated levels of a trio of markers, specifically IL-6, IL-8, and TNF-α, and mortality [10].

Despite these concerns, our group and others have found that PLWH co-infected with SARS-CoV-2 have less severe disease than their HIV-uninfected counterparts [11,12]. These results are difficult to explain, given the paucity of information about the immunological profiles of PLWH co-infected with SARS-CoV-2 in South Africa. Since a hallmark of COVID-19 is an exacerbated immune response, we hypothesized that pre-existing T-cell dysregulation in PLWH might attenuate the development of a hyperinflammatory state with SARS-CoV-2 infection. It needs to be considered that PLWH are not a homogenous group, and variable immune responses can be expected depending on the CD4+ T-cell count (below versus equal to or above 200 cells/mm^3^) and presence of viremia (detectable or undetectable HIV viral load [VL]). Therefore, this study explored whether differences existed in the T-cell phenotypes and systemic cytokine and chemokine profiles of patients admitted to a hospital with COVID-19 with and without HIV co-infection, as well as among PLWH based on their CD4+ T-cell count and HIV VL at admission.

## 2. Materials and Methods

### 2.1. Study Population

Consecutive COVID-19 patients admitted to the Steve Biko Academic and Tshwane District Hospitals from May 2020 to December 2021 who met the inclusion criteria were recruited. The criteria included SARS-CoV-2 polymerase chain reaction (PCR) positivity, an age of 18 years or older, and being willing and able to provide informed consent. Blood samples were collected in EDTA-containing tubes on the first day of admission (V1) before starting COVID-19 treatment. The participants received corticosteroids (prednisone or dexamethasone) and antibiotics (amoxicillin–clavulanic acid and/or azithromycin) post-admission. Flow cytometry was performed on the same day as the blood draw, and plasma was stored at −80 °C until use. Blood samples were also collected ±7 days post-admission (V2), followed by flow cytometry. Routine pathology results were extracted from the National Health Laboratory Service (NHLS) Trakcare Database of South Africa.

Ten HIV-negative controls were recruited from the University of Pretoria staff and students, and 19 PLWH control participants were recruited from The Steve Biko Academic Hospital in 2020 before the COVID-19 pandemic. Rapid antibody tests confirmed the absence of prior SARS-CoV-2 infection in these participants.

The study evaluated the following four groups: PLWH hospitalized with COVID-19 (COVID+ PLWH), PLWH controls without COVID-19 (Control PLWH), people living without HIV hospitalized with COVID-19 (COVID+ PLWOH), and control people living without HIV without COVID-19 (Control PLWOH). Immune responses are variable in PLWH depending on their viremia and CD4+ T-cell count, thus, the COVID+ PLWH and Control PLWH groups were subdivided by a CD4+ T-cell count equal to or above or below 200 cells/mm^3^ (COVID+ PLWH were subdivided into COVID+ CD4+ T-cells of ≥200 and COVID+ CD4+ T-cells of <200 cells/mm^3^, while Control PLWH were divided into Control CD4+ T-cells of ≥200 and Control CD4+ T-cells of <200 cells/mm^3^), and based on their HIV VL (COVID+ PLWH were divided into “COVID+ detectable VL” and “COVID+ undetectable VL”, while Control PLWH were divided into “Control detectable VL” and “Control undetectable VL”). The study was approved by the Health Sciences Research Ethics Committee, University of Pretoria (ref. 247/2020).

### 2.2. T-Cell Flow Cytometry

T-cell phenotypes were investigated using a DuraClone T-cell subset kit (Beckman Coulter Inc., Brea, CA, USA). Dry antibody reagents included the following markers: CD45RO, CD45RA, CD3, CD4, CD8, CD28, CD27, C-C chemokine receptor type 7 (CCR7), PD-1, and CD57. Fifty microliters of whole blood were added to the DuraClone T-cell subset tube. The protocol was followed as per the manufacturer’s instructions. Acquisition was performed using a CytoFlex flow cytometer (Beckman Coulter Inc., Brea, CA, USA). Detailed flow cytometry methods are provided in the Supplementary Materials (Section S2.1).

### 2.3. Biomarker Analysis

#### 2.3.1. Cytokine and Chemokine Quantification

The concentrations of systemic cytokines and chemokines were determined in the stored plasma samples using Bio-Plex Human Cytokine/Chemokine Panel kits and Bio-Plex Human Treg Cytokine Panel kits (Bio-Rad Laboratories, Inc., Hercules, CA, USA). The plasma samples were diluted four-fold and the experimental procedure was followed as per the manufacturer’s instructions. Analysis was performed on a Bio-Plex Suspension Array platform (Bio-Rad Laboratories, Inc., Hercules, CA, USA). Bio-Plex Manager Software 6.0 was used for bead acquisition and analysis of the median fluorescence intensity. The results are presented as picograms per milliliter (pg/mL).

#### 2.3.2. Transforming Growth Factor-β1 Measurement

Transforming growth factor β1 (TGF-β1) levels were determined using the Human TGF-β1 ELISA kit (E-EL-0162, Elabscience Biotechnology, Inc., Houston, TX, USA). Prior to analysis, latent TGF-β1 was activated to the immunoreactive form by adding 40 µL 1N hydrochloric acid to 240 µL plasma (diluted eight-fold). The experimental procedure was followed as per the manufacturer’s instructions. The samples were assayed immediately, and the results are expressed in nanograms (ng)/mL.

### 2.4. Statistical Analysis

Clinical information was captured from patient files and entered by two independent researchers into Excel spreadsheets. These sheets were compared to identify any data entry errors. The results of routine laboratory tests were obtained from the NHLS. Data were exported to Stata 17 for analysis, assessed for their distribution, and appropriate tests were applied. Pearson’s chi-square and Fisher’s exact tests were used to compare categorical variables, depending on the expected cell frequency. Due to the non-normal distribution of the data, the Kruskal–Wallis test with a post hoc Dunn’s test was used to compare continuous variables between groups. The Wilcoxon test for paired samples with Bonferroni correction was used for univariate comparisons of paired V1 and V2 continuous variables. Stepwise, backward, multivariable logistic regression analyses were used to examine associations with the outcome variable after the appropriate transformation of predictor variables, as determined by the gladder command. The assumptions used were as follows: (1) a binary outcome variable with only two possible response categories; (2) a linear relationship between the log-odds of the outcome and independent variables; (3) independent errors (i.e., no obvious clustering); and (4) no severe multicollinearity as determined by the variance inflation factor. Spearman’s correlation test, with Bonferroni correction for multiple comparisons, was used to assess correlations between continuous variables.

## 3. Results

### 3.1. Demographic and Clinical Details of Patients

This study recruited 178 patients hospitalized with COVID-19, excluding four without a positive SARS-CoV-2 PCR test. Among the participants, 37 (21%) were PLWH who were significantly younger and more likely to be female (COVID+ PLWH: 70% female and 30% male vs. COVID+ PLWOH: 41% female and 59% male) (Table 1). Comorbidity and outcome data were available for 122/137 and 134/137 people without HIV and with COVID-19, respectively. PLWH had a lower prevalence of diabetes, but a higher incidence of *Mycobacterium tuberculosis* (MTB) infection. The MTB status of one PLWH was unknown. Among the PLWH, 26/37 (70.3%) were on ART and 19/37 (51.4%) had an undetectable HIV viral load (<20 copies/mL) at admission. The CD4+ T-cell counts were known in 36/37 (97.3%) PLWH, with nearly half (17/36, 47.22%) having a CD4+ T-cell count of <200 cells/mm^3^. PLWH exhibited a lower fraction of inspired oxygen (FiO_2_) and a higher ratio of the partial pressure of oxygen in the arterial blood (PaO_2_) to FiO_2_ (P/F ratio)—used to classify the severity of ARDS—at admission, indicating a reduced need for oxygen therapy. The P/F ratio was only available for 83/174 (47.7%) patients at admission. Albeit not significant, a lower proportion of PLWH were admitted with a P/F ratio below the critical threshold of 200 (COVID+ PLWH: 13/21 [61.9%] vs. COVID+ PLWOH: 44/61 [72.13%], *p* = 0.380). Additionally, PLWH had lower blood ferritin and procalcitonin (PCT) levels than people without HIV, while the CRP levels were elevated in both groups, with no significant difference between patient groups.

The mean age of the PLWH controls (SARS-CoV-2 negative) was 47 ± 14 years, with 12/19 (63%) being female and 7/19 (37%) being male; only two participants were not undergoing ART. The median CD4+ T-cell count for the control participants was 246 (IQR: 162–671) cells/mm^3^, with 8/19 (42%) having a CD4+ T-cell count of <200 cells/mm^3^. The median VL was 20 (20–19,900) copies/mL, and 9/19 (47%) had detectable VL. The mean age of the control participants without HIV was 44 ± 9 years, with 3/10 (30%) being female and 7/10 (70%) being male. Flow cytometry data were unavailable for 1/37 PLWH and 20/137 people without HIV admitted with COVID-19.

### 3.2. Visit 1 CD4+ and CD8+ T-Cell Subset Differences Between People Living With and Without HIV Hospitalized with COVID-19

PLWH with COVID-19 had lower percentages of CD4+ T-cells (*p* < 0.001) and higher percentages of CD8+ T-cells (*p* < 0.001) than those without HIV. The median CD4:CD8 ratio was significantly lower in PLWH, at 1.03 (IQR: 0.21–1.69) vs. 2.41 (IQR: 1.37–4.1) (*p* < 0.001). PLWH had lower percentages of double-positive (DP) (*p* < 0.001) and higher percentages of double-negative (DN) (*p* = 0.015) T-cells than those without HIV. No differences were found in terms of CD4+, CD8+, DP, and DN T-cells between the patient groups and their respective controls, indicating that these differences were not related to COVID-19, but rather to HIV.

**Figure 1:**
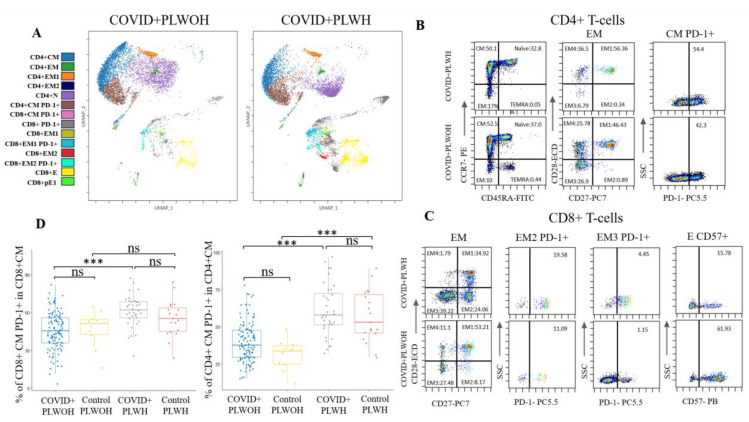
Visit 1 differences in T-cell populations between people living with and without HIV hospitalized with COVID-19. (**A**) Representative UMAP plots of people living with and without HIV at admission with COVID-19. (**B**) Representative dot plots of people living with and without HIV showing differences in CD4+ T-cell subsets, CD4+ EM subsets, and CM PD-1+ expression. (**C**) Representative dot plots of people living with and without HIV showing differences in CD8+ EM subsets and PD-1 expression in CD8+ EM2 and EM3 populations, as well as TEMRA end-stage effectors expressing CD57. (**D**) COVID+ PLWH had a higher percentage of CD4+ and CD8+ CM T-cells expressing PD-1. Abbreviations: central memory (CM), control PLWH without COVID-19 (Control PLWH), control people living without HIV without COVID-19 (Control PLWOH), double negative (DN), double positive (DP), end-stage effector (E), effector memory (EM), not significant (ns), pre-effector 1 (pE1), PLWH hospitalized with COVID-19 (COVID+ PLWH), people living without HIV hospitalized with COVID-19 (COVID+ PLWOH), programmed cell death protein 1 (PD-1), and terminally differentiated T-cells re-expressing CD45RA (TEMRA). The Kruskal–Wallis test with post hoc Dunn’s test was used to compare continuous variables between groups. *p*-value: ***: <0.001.

In terms of T-cell memory subsets, PLWH had lower percentages of CD4+ central memory (CM) T-cells (*p* = 0.025), but higher percentages of CD4+ CM T-cells expressing PD-1 (*p* < 0.001) at V1 (Figure 1). In contrast, PLWH had higher percentages of CD4+ effector memory (EM) T-cells (*p* = 0.002), but significantly lower percentages of CD4+ EM2 T-cells (*p* = 0.018) (Figure 1).

PLWH had a higher percentage of CD8+ CM T-cells expressing PD-1 (*p* < 0.001) (Figure 1). Among the EM subsets, PLWH had lower percentages of CD8+ EM1 (*p* < 0.001), significantly lower percentages of CD8+ EM1 PD-1+ (*p* = 0.012), and higher percentages of CD8+ EM2 (*p* < 0.001), CD8+ EM2 PD-1+ (*p* < 0.001), and CD8+ EM3 PD-1+ (*p* = 0.008) T-cells (Figure 1). PLWH had lower percentages of CD8+ terminally differentiated T-cells re-expressing CD45RA (TEMRA) pre-effector 1 (*p* = 0.027) and end-stage effector T-cells expressing the immunosenescent marker CD57 (*p* = 0.006).

Supplementary Table S1 shows the comparisons with the respective control groups.

### 3.3. Visit 1 Cytokine and Chemokine Differences Between People Living With and Without HIV Hospitalized with COVID-19

Differences in the levels of cytokines and chemokines are shown in Supplementary Tables S2 and S3. Based on the prominence of IL-6 and regulatory cytokines (IL-2, IL-10, and its counterpart IL-19, as well as IL-12, IL-20, IL-26, IL-27, IL-28A, IL-29, IL-35, and TGF-β1), these were included in the regression model. The CD8+ EM2 T-cell population was negatively correlated with IL-35 in both PLWH (r[35] = −0.435, *p* = 0.009) and those without HIV (r[102] = −0.230, *p* = 0.020).

### 3.4. Logistic Regression of Visit 1 T-Cell Populations, Cytokines, and Chemokines by HIV Status

A stepwise backward logistic multivariable regression model (Table 2), correcting for sex and age by HIV status, revealed that PLWH had significantly higher percentages of CD4+ CM PD-1+, CD8+ EM2, and CD8+ EM4 CD57+, as well as higher concentrations of IL-35 and lower concentrations of IL-19 at V1.

### 3.5. Differences in T-Cell Profiles, Cytokines, and Chemokines at the Time of Hospitalization with COVID-19 in PLWH According to HIV Viral Load and CD4+ T-Cell Count

**Figure 2:**
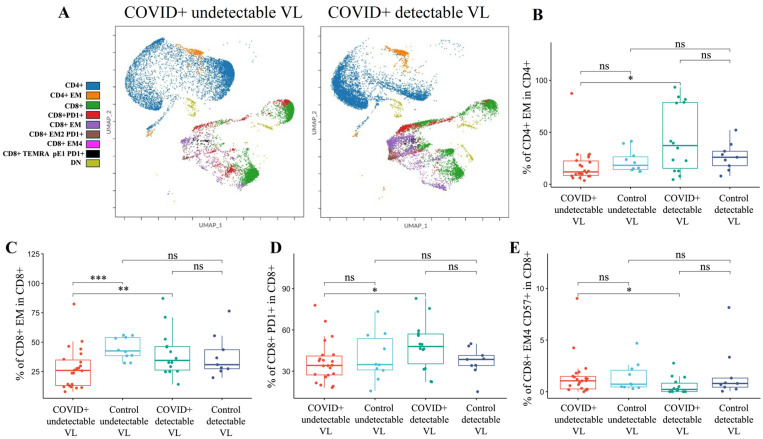
T-cell subsets comparing PLWH with undetectable and detectable HIV viral loads upon hospitalization with COVID-19, as well as their respective controls. (**A**) Representative UMAP plots of the T-cell populations of PLWH with undetectable and detectable VLs at admission with COVID-19. (**B**) PLWH with a detectable VL with COVID-19 had significantly higher percentages of CD4+ EM T-cells compared to PLWH with an undetectable VL at admission. (**C**) PLWH with a detectable VL with COVID-19 had significantly higher percentages of CD8+ EM T-cells compared to PLWH with an undetectable VL. PLWH with a detectable VL admitted with COVID-19 had significantly lower percentages of CD8+ EM T-cells compared to PLWH with undetectable VL controls. (**D**) PLWH with a detectable VL with COVID-19 had significantly higher percentages of CD8+ PD-1+ T-cells than PLWH with an undetectable VL at admission. (**E**) PLWH with a detectable VL with COVID-19 had significantly lower percentages of CD8+ EM4 CD57+ T-cells compared to PLWH with an undetectable VL at admission. Abbreviations: central memory (CM), double negative (DN), effector memory (EM), not significant (ns), programmed cell death protein 1 (PD-1), pre-effector (pE), terminally differentiated effector memory T-cells re-expressing CD45RA (TEMRA), and viral load (VL). The Kruskal–Wallis test with post hoc Dunn’s test was used to compare continuous variables between groups. *p*-value: *: <0.05, **: <0.01, ***: <0.001.

Multiple differences in T-cell populations were found between PLWH with undetectable and detectable HIV VLs on admission to the hospital with COVID-19 (Figure 2). COVID-19+ PLWH with a detectable VL (*n* = 18) had significantly lower percentages of CD4+ (*p* = 0.008), CD8+ EM4 CD57+ (*p* = 0.013), and CD8+ TEMRA pE1 T-cells expressing PD-1 (*p* = 0.005) than COVID+ PLWH with an undetectable VL (*n* = 19). On the other hand, PLWH with a detectable VL had higher percentages of DN (*p* = 0.017), CD8+ (*p* = 0.024), CD4+ EM (*p* = 0.019), CD8+ EM (*p* = 0.005), CD8+ PD-1+ (*p* = 0.016), and CD8+ EM2 PD-1+ (*p* = 0.039) T-cells.

**Figure 3:**
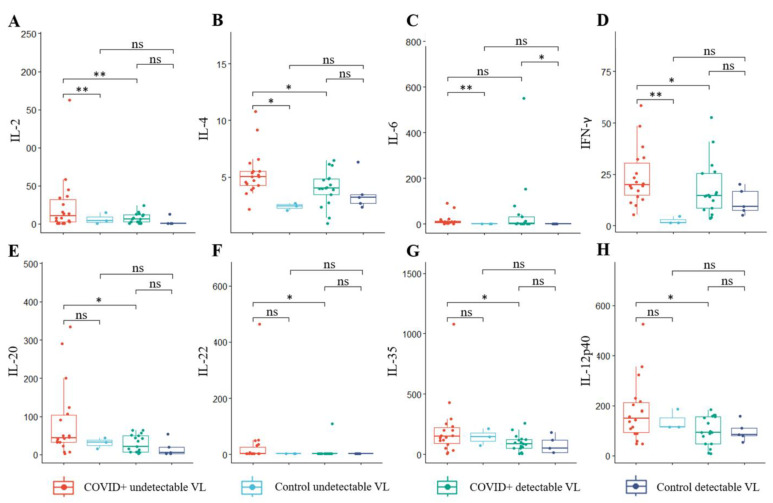
Comparison of cytokine concentrations in PLWH hospitalized with COVID-19 with detectable and undetectable HIV VLs and their respective controls. (**A**) IL-2 concentrations were significantly higher in PLWH with an undetectable VL than in both PLWH with a detectable VL at admission and PLWH with undetectable VL controls. (**B**) IL-4 concentrations were significantly higher in PLWH with an undetectable VL than in both PLWH with a detectable VL at admission and PLWH with undetectable VL controls. (**C**) IL-6 concentrations were higher in both patient groups compared to their respective controls. (**D**) IFN-γ concentrations were significantly higher in PLWH with an undetectable VL than in both PLWH with a detectable VL at admission and PLWH with undetectable VL controls. (**E**–**H**) Concentrations of IL-20, IL-22, IL-35, and IL-12p40 were significantly higher in PLWH admitted with COVID-19 with an undetectable VL than in PLWH with a detectable VL. No significant differences were found between the patient groups and the respective control groups. Abbreviations: interleukin (IL), interferon (IFN), not significant (ns). The Kruskal–Wallis test with post hoc Dunn’s test was used to compare continuous variables between groups. Results are presented as median and interquartile range (IQR). *p*-value: *: <0.05, **: <0.01.

PLWH with COVID-19 with a detectable VL had significantly lower concentrations of IL-2, IL-4, IFN-γ, IL-20, IL-22, IL-35, and IL-12p40 than PLWH with COVID-19 with an undetectable VL (Figure 3). IL-6 concentrations were higher in both COVID-19 patient groups compared to the respective control cohorts. PLWH with COVID-19 with an undetectable VL had higher concentrations of IL-2, IL-4, and IFN-γ than PLWH without COVID-19 with a detectable VL.

**Figure 4:**
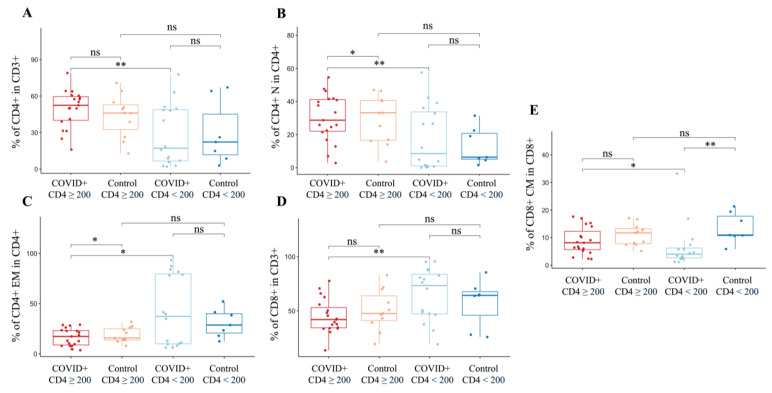
T-cell phenotypes of PLWH hospitalized with COVID-19 with CD4+ T-cell counts < or ≥200 cells/mm^3^. (**A**) PLWH with a CD4+ T-cell count < 200 cells/mm^3^ had significantly lower percentages of CD4+ T-cells than PLWH with a CD4+ T-cell count ≥ 200 cells/mm^3^. (**B**) PLWH with a CD4+ T-cell count < 200 cells/mm^3^ had significantly lower percentages of CD4+ N T-cells than PLWH with a CD4+ T-cell count ≥ 200 cells/mm^3^. PLWH with a CD4+ T-cell count ≥ 200 cells/mm^3^ also had a significantly higher percentage of CD4+ N T-cells than PLWH controls with a CD4+ T-cell count ≥ 200 cells/mm^3^. (**C**) PLWH with a CD4+ T-cell count ≥ 200 cells/mm^3^ had significantly lower percentages of CD4+ EM T-cells than both PLWH with a CD4+ T-cell count < 200 cells/mm^3^ and the respective PLWH controls without COVID-19. (**D**) PLWH with a CD4+ T-cell count < 200 cells/mm^3^ had significantly lower percentages of CD8+ T-cells than PLWH with a CD4+ T-cell count ≥ 200 cells/mm^3^. (**E**) PLWH with a CD4+ T-cell count of <200 cells/mm^3^ with COVID-19 had significantly lower percentages of CD8+ CM T-cells than both PLWH with a CD4+ T-cell count of ≥200 cells/mm^3^ and control PLWH with a CD4+ T-cell count of <200 cells/mm^3^. Abbreviations: central memory (CM), effector memory (EM), naïve (N), and not significant (ns). The Kruskal–Wallis test with post hoc Dunn’s test was used to compare continuous variables between groups. *p*-value: *: <0.05, **: <0.01.

Ten (55.56%) PLWH with a detectable VL also had a CD4+ count of <200 cells/mm^3^. PLWH with a CD4+ T-cell count of < 200 cells/mm^3^ (*n* = 17) had lower percentages of CD4+ (*p* = 0.002) and higher percentages of CD8+ T-cells than those with counts of ≥200 cells/mm^3^ (*n* = 19) (*p* = 0.004) (Figure 4). These individuals also had significantly lower percentages of CD4+ N (*p* = 0.009) and CD8+ CM (*p* = 0.025) and higher percentages of CD4+ EM (*p* = 0.020) T-cells.

PLWH with a CD4+ T-cell count of <200 cells/mm^3^ had significantly higher concentrations of IL-6 (13.18 [IQR: 5.39–72.3] vs. 4.32 [IQR: 1.25–7.28], *p* = 0.009) and significantly lower concentrations of IL-29 (18.995 [IQR: 6.99–33.08] vs. 50.19 [IQR: 27.02–57.47], *p* = 0.016) than PLWH with a CD4+ T-cell count of ≥200 cells/mm^3^ (Supplementary Tables S4 and S5).

PLWH admitted with COVID-19 that were not undergoing ART 11/37 (29.7%) had lower concentrations of IL-2 (COVID+ PLWH not on ART median: 4.54 [IQR: 0.99–8.09] vs. COVID+ PLWH on ART median: 9.00 [IQR: 3.21–14.79], *p* = 0.018), IL-10 (COVID+ PLWH not on ART median: 7.77 [IQR: 0–16.11] vs. COVID+ PLWH on ART median: 16.84 [IQR: 0.85–32.83], *p* = 0.047), and TGF-β1 (COVID+ PLWH not on ART median: 5.58 [IQR: 0.52–10.64] vs. COVID+ PLWH on ART median: 9.84 [IQR: 3.06–16.62], *p* = 0.060). No significant differences could be found in terms of IFN-γ concentrations between PLWH with COVID-19 that were undergoing ART and those not receiving ART at the time of admission (COVID+ PLWH not on ART median: 14.76 [IQR: 6.01–23.51] vs. COVID+ PLWH on ART median: 19.47 [IQR: 1.26–37.68], *p* = 0.810).

### 3.6. Visit 2 Differences in CD4+ and CD8+ T-Cell Profiles Between People Living With and Without HIV Hospitalized with COVID-19

Blood samples were available from 69/174 (39.65%) COVID-19 patients at V2. There was no differences in sex between PLWH (7/17, 41% male) and those without HIV (28/52, 54% male) at V2 (*p* = 0.364). Oxygen saturation improved between V1 and V2, as indicated by FiO_2_ (*p* < 0.001) and PaO_2_ (*p* = 0.034). CRP levels were lower at V2 than at admission, although not significantly so (V1 median: 124 mg/L [70–216] vs. V2 median: 65 mg/L [26–218], *p* = 0.452).

Corticosteroid use varied across pandemic waves, with no differences observed in the administration of corticosteroids between PLWH and those without HIV (*p* = 0.319) or in the proportion of people with or without HIV treated across the four waves (*p* = 0.187). Antibiotics were routinely administered, with no differences between PLWH and those without HIV (*p* = 0.514). South Africa lacked access to antiviral medication for COVID-19 during the duration of the study. Comparing the T-cell populations at V2, patients who received corticosteroids had lower percentages of CD8+ end-stage effectors co-expressing CD57 and PD-1 (*p* = 0.024) and CD8+ EM4 PD-1+ T-cells (*p* = 0.031) and higher percentages of CD8+ pre-effector 1 T-cells (*p* = 0.011).

**Figure 5:**
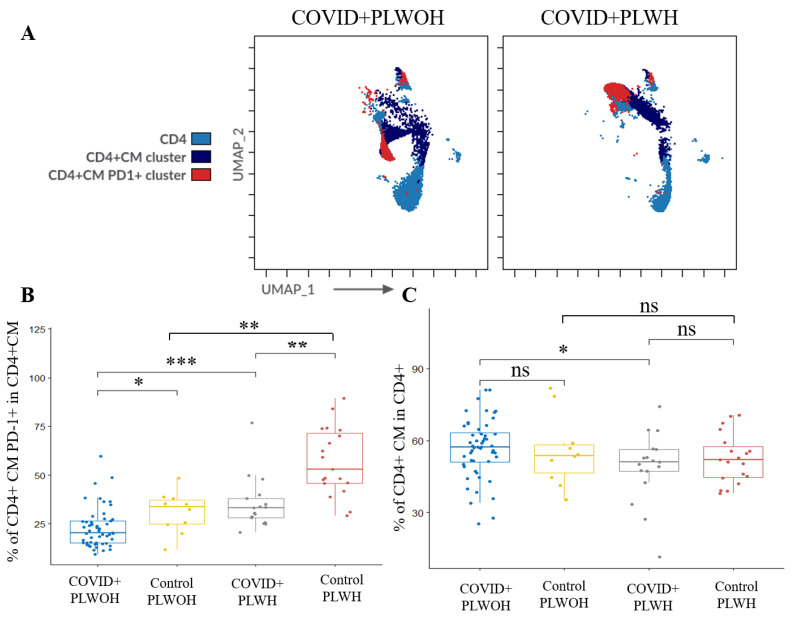
Comparison of CD4+ T-cell phenotypes at Visit 2 between people living with and without HIV hospitalized with COVID-19 and their respective controls. (**A**) Representative UMAP plots of CD4+ T-cell populations in people living with and without HIV at Visit 2. (**B**) COVID+ PLWH had significantly higher percentages of CD4+ CM PD-1+ T-cells when compared to COVID+ PLWOH at V2. Both patient groups (COVID+ PLWH and COVID+ PLWOH) had lower percentages of CD4+ CM PD-1+ T-cells when compared to their respective controls not admitted with COVID-19. (**C**) COVID+ PLWH had significantly lower percentages of CD4+ CM T-cells overall compared to COVID+ PLWOH at Visit 2. No significant difference was found between the patient groups and respective control groups in terms of the percentage of CD4+ CM T-cells. Abbreviations: central memory (CM), control people living without HIV (PLWOH) without COVID-19 (Control PLWOH), control PLWH without COVID-19 (Control PLWH), effector memory (EM), not significant (ns), PLWH hospitalized with COVID-19 (COVID+ PLWH), PLWOH hospitalized with COVID-19 (COVID+ PLWOH), and programmed cell death protein 1 (PD-1). The Kruskal–Wallis test with post hoc Dunn’s test was used to compare continuous variables between groups. *p*-value: *: <0.05, **: <0.01, ***: <0.001.

**Figure 6:**
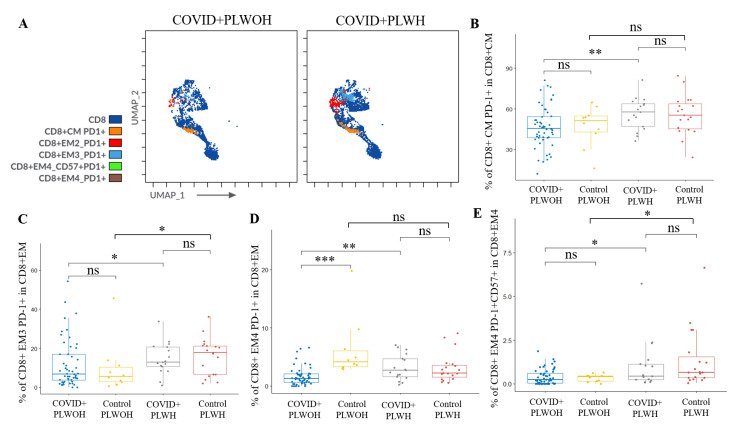
Comparison of CD8+ T-cell phenotypes at Visit 2 between people living with and without HIV hospitalized with COVID-19 and their respective controls. (**A**) Representative UMAP plots of CD8+ T-cell populations of people living with and without HIV at Visit 2. (**B**) COVID+ PLWH had significantly higher percentages of CD8+ CM PD-1+ T-cells than COVID+ PLWOH. No significant differences were found between patient groups and the respective controls in terms of the percentage of CD8+ CM PD-1+ T-cell population. (**C**) COVID+ PLWH admitted with COVID-19 had significantly higher percentages of CD8+ EM3 PD-1+ T-cells than COVID+ PLWOH admitted with COVID-19. The same difference was found between control participants; Control PLWH had higher percentages of CD8+ EM3 PD-1+ T-cells than Control PLWOH. (**D**) COVID+ PLWOH had significantly lower percentages of CD8+ EM4 PD-1+ when compared to both COVID+ PLWH and their respective control group without COVID-19. (**E**) COVID+ PLWH admitted with COVID-19 had significantly higher percentages of CD8+ EM4 PD-1+ CD57+ T-cells when compared to COVID+ PLWOH. The same difference was found between control participants: Control PLWH had higher percentages of CD8+ EM4 PD-1+ CD57+ T-cells than Control PLWOH. Abbreviations: central memory (CM), control PLWH without COVID-19 (Control PLWH), control people living without HIV without COVID-19 (Control PLWOH), effector memory (EM), not significant (ns), people living without HIV (PLWOH) hospitalized with COVID-19 (COVID+ PLWOH), PLWH hospitalized with COVID-19 (COVID+ PLWH), and programmed cell death protein 1 (PD-1). The Kruskal–Wallis test with post hoc Dunn’s test was used to compare continuous variables between groups. *p*-value: *: <0.05, **: <0.01, ***: <0.001.

PLWH had higher percentages of DN, CD8+, and CD8+ CM PD-1+ T-cells and lower percentages of CD4+ and CD4+ CM T-cells than those without HIV. However, a higher percentage of CD4+ CM T-cells expressed PD-1 in PLWH (*p* < 0.001) (Figure 5). PLWH had higher percentages of CD8+ T-cells expressing PD-1 within the EM3 (*p* = 0.033) and EM4 (*p* = 0.003) subsets, as well as higher percentages of CD8+ EM4 cells co-expressing CD57 and PD-1 (*p* = 0.016) (Figure 6) (Supplementary Table S10).

Both people with and without HIV showed differences in certain T-cell populations between the control and COVID-19 groups. CD4+ EM1 PD-1+ was higher in the COVID-19 group compared to the control cohort (*p* < 0.001). Populations that were lower in the COVID-19 groups compared to the control groups were CD8+ EM4 (COVID-19 + PLWH vs. PLWH Controls, *p* = 0.075; COVID-19 + PLWOH vs. Controls PLWOH, *p* = 0.001, PLWH Control vs. Control PLWOH, *p* = 0.041) and CD8+ CM (COVID-19 + PLWH vs. PLWH Controls, *p* = 0.003; COVID-19 + PLWOH vs. Controls PLWOH, *p* = 0.013, PLWH Control vs. Control PLWOH, *p* = 0.346) T-cells.

Stepwise backward multivariable logistic regression, adjusting for age, revealed differences in the T-cell populations of people living with and without HIV at V2 (Table 3). PLWH hospitalized with COVID-19 had higher percentages of CD4+ CM PD-1+, CD8+, and CD8+ EM4 T-cells co-expressing CD57 and PD-1, and lower percentages of the CD8+ EM subset.

## 4. Discussion

Previous reports from our group and others have indicated that PLWH exhibit fewer inflammatory markers and less severe disease upon hospital admission for COVID-19 [11,12,13]. This study aimed to investigate differences in T-cell phenotypes and systemic cytokine profiles to explain these observations. The P/F ratio is currently used to identify acute respiratory failure in patients with COVID-19 and adults with ARDS [14]. A P/F ratio below 200 is considered to be a critical threshold, indicating a high risk of developing ARDS and respiratory failure [14]. In the current study, PLWH exhibited a higher P/F ratio on admission, indicating a lower oxygen demand. Together with their reduced levels of ferritin and PCT, both markers of acute-phase response to inflammation, these findings are indicative of milder COVID-19.

In line with the current study, multiple studies have been published that have reported that PLWH with an undetectable HIV VL undergoing ART have similar clinical presentations of COVID-19 and are not at an increased risk of morbidity or mortality when compared to those without HIV [15,16,17,18]. A study conducted in South Africa by Venturas et al. compared the outcomes of COVID-19 in people living with and without HIV admitted to a tertiary referral center in Johannesburg [11]. Three hundred and eighty-four adult patients admitted to general wards and intensive care unit (ICU) wards between 6 March and 11 September 2020 were included in the study [11]. Of these, 108/384 (28%) individuals were PLWH and 276/384 (72%) were HIV-negative. The median CD4+ T-cell count for PLWH was 210 (IQR: 180–339) cells/mm^3^ [11]. These authors found that PLWH admitted to hospital with COVID-19 were frequently younger than those without HIV [11]. As was the case in our study, Venturas et al. concluded that there was no increased risk of severe disease or mortality in PLWH when compared to those living without HIV (15% vs. 20%) [11].

A study conducted in Spain found that PLWH well-controlled on ART admitted with COVID-19, despite being older, had lower serum CRP levels than those living without HIV with COVID-19 [15]. During this study, 61.9% of PLWH and 78.4% of people without HIV received oxygen. Accordingly, similar to the current study, a lower percentage of PLWH in the above-mentioned cohort required oxygen therapy during their hospital stay, and even fewer required mechanical ventilation (9.5% vs. 23.3% respectively) [15]. While Hadi et al. found that the COVID-19 crude mortality was higher in PLWH when compared to those living without HIV, propensity-matched analysis matching for co-morbidities revealed no significant differences, indicating that it is not HIV itself, but the high burden of co-morbidities that frequently accompanies this condition that drives the higher risk of COVID-19 mortality [17].

Another study from the USA found that the highest proportion of PLWH (1638 cases) presented to the hospital with mild disease (47.6%), followed by moderate disease (38.3%), while only 15.3% presented or developed severe disease which required ICU admission or resulted in death [19].

Our findings contrast with other studies that have reported more severe disease and higher inflammation in PLWH with COVID-19. Augello et al. found that PLWH had worse respiratory function, indicated by lower P/F ratios and higher inflammatory cytokine levels [20]. This discrepancy likely stems from the following demographic differences: our cohort consisted mainly of younger women (70% female, mean age of 46 years), whereas Augello et al.’s cohort comprised older men (22.2% female, median age of 60 years, 77.8% male, median age of 60 years) [20]. Peluso et al. reported higher systemic inflammation in PLWH with post-acute sequelae of SARS-CoV-2 than in those living without HIV, with their study also primarily involving male participants (95%) [21]. A meta-analysis published in March 2024, investigating the severity of various forms of COVID-19 in PLWH, as opposed to people living without HIV, including 13 studies from the Americas, Europe, and Asia, found that these regions reported a greater proportion of men affected than women [22]. On the other hand, studies conducted in Africa investigating PLWH co-infected with SARS-CoV-2 had similar age and sex distributions as those described in the current study—Nkosi et al. (median age: 40.5 [IQR 30–51.75], 29.16% male), Mnguni et al. (median age: 46 [IQR 37–54], 29.1% male), and Venturas et al. (median age: 45 [IQR 38–56] 50% male) [11,23,24]. An older age and male sex are established risk factors for severe disease and mortality in COVID-19 [25]. This underscores regional demographic differences among PLWH and the necessity of considering these factors when interpreting data.

After adjusting for sex and age, the only significantly different T-cell populations at hospital admission between people with and without HIV were higher percentages of CD4+ CM expressing PD-1, CD8+ EM2, and CD8+ EM4 expressing the senescence marker CD57 in PLWH.

The CD4+ CM PD-1+ population was significantly higher in PLWH with COVID-19 at both time points. Persistent antigenic stimulation during chronic viral infection is associated with the T-cell expression of inhibitory immune checkpoint markers such as PD-1 that downregulate immune responses [26]. In the setting of chronic HIV infection, a high expression of PD-1 is related to impaired immunologic function, despite prolonged HIV viral suppression. An important caveat, however, is that, although the total PD-1 expression on T-cells has been used to define an exhausted T-cell phenotype in the literature, T-cells rapidly express PD-1 during T-cell receptor-mediated antigen activation [26,27]. Once the infection is cleared, expression levels decrease [26,27]. As such, the total PD-1 expression alone is not necessarily a specific marker for a subset of exhausted T-cells in the setting of persistent antigen stimulation, and should ideally be interpreted together with the expression of its ligands on other immune cells [26]. PD-1 expression on circulating CD4+ T-cells contributes to the transition from asymptomatic to symptomatic SARS-CoV-2 infection [9]. Niedźwiedzka-Rystwej et al. observed increased PD-1 expression on T-cells in patients in the ICU and those who died [28]. HIV also upregulates PD-1 on T-cells, particularly CD4+ CM T-cells [29]. We hypothesize that the CD4+ CM PD-1+ population in PLWH is a pre-existing population due to HIV, which may be further expanded by SARS-CoV-2. PD-1 expression reduces T-cell proliferation and cytokine production, thereby mitigating a hyperinflammatory response [29].

HIV alters T-cell differentiation and maturation. In this context, Mojumdar et al. found that PLWH have an over-representation of CD8+ EM2 T-cells (CD27+ CD28−) [30]. This skewed CD8+ T-cell maturation could possibly be a mechanism utilized by HIV to prevent CD8+ T-cells from gaining full effector function, which occurs early in HIV infection and is irreversible with ART [30]. EM2 CD8+ T-cells have a lower cytotoxic activity and cytokine production compared to EM3 T-cells [31]. EM3 T-cells more closely resemble CD8+ TEMRA cells [32]. Despite their poor proliferation, CD8+ EM2 T-cells effectively eliminate infected cells by producing perforin and granulysin and producing cytokines that target viral clearance [31,32]. Burnett et al. correlated the CD8+ EM2 subset with better outcomes and ventilation resolution in patients with COVID-19 [33].

Upon hospitalization with COVID-19, PLWH had elevated IL-35 levels compared to those without HIV. IL-35 primarily originates from regulatory T-cells (Tregs), followed by tolerogenic dendritic cells (DCs), regulatory B-cells, and macrophages [34]. IL-35 is a recently discovered anti-inflammatory cytokine which promotes immune suppression by inhibiting effector cell proliferation via cell cycle arrest, expanding Tregs, and modulating T-cell differentiation [34,35]. The exact mechanism of IL-35-induced cell cycle arrest in effector cells remains unclear [34]. The production of IL-35 in tissues is induced by inflammatory stimuli and then transcribed by smooth muscle cells and endothelial cells [35]. IL-35 dampens the inflammatory response through its interaction with the IL-35 receptor (IL-35R), which results in the phosphorylation of Janus kinase (JAK)2/signal transducers and activators of transcription (STAT)1/4 signaling pathways, which, in turn, enhances the inhibitory effect of leukocyte-associated immunoglobulin-like receptor 1 [36]. IL-35 can also suppress monocyte-derived DCs by activating STAT1/3 pathways, while simultaneously inhibiting the nuclear factor kappa B (NF**-**_Κ_B) and p38 mitogen-activated protein kinase (MAPK) pathways, thereby reducing pro-inflammatory signaling [37]. The inhibitory effects of IL-35 have been described in studies that have investigated bacterial or parasitic infections and chronic inflammatory conditions [35]. A recent study showed that IL-35 can reduce airway eosinophilia through the inhibition of eosinophil-attracting chemokines (CCL24 and CCL11) and concluded that IL-35 could be a treatment option for reducing the recruitment of eosinophils into tissues in disorders such as asthma [37]. IL-35 has also been linked to chronic obstructive pulmonary disease (COPD), in which Himani et al. found lower concentrations of IL-35 in patients who developed COPD [38].

In terms of SARS-CoV-2 infection, a study by AL-Khikani et al. found significantly higher concentrations of IL-35 in patients with severe COVID-19 compared to healthy controls [37]. The group also found significant positive correlations between IL-35 and blood glucose levels, as well as creatinine, which they proposed indicates the protective effect of IL-35 in controlling the inflammatory response in the acute kidney injury seen in patients with severe COVID-19 [37]. Mohammed et al. also reported that the concentrations of IL-35 differed between patients with COVID-19 and healthy controls. In addition, patients showed strong positive correlations between IL-35, the inflammatory cytokine IL-6, and CRP [35]. Other studies found upregulated IL-35 and IL-10 transcripts in the Tregs of patients with severe COVID-19 [39]. These mechanisms most likely developed to counteract the immune hyperactivation caused by SARS-CoV-2 infection [40].

IL-35 has also been implicated in other viral infections. Li et al. demonstrated that IL-35 is highly expressed in hepatitis B virus (HBV)-specific CD4+ T-cells and plays a very important role in the inhibition of the cellular immune response during chronic HBV infection [41]. In vivo*,* these authors found that IL-35 suppressed the proliferation of antigen-specific cytotoxic T-cells, which, in turn, led to lower IFN-γ production [41]. In ex vivo experiments, Li and colleagues reported the decreased proliferation of naïve effector T-cells (CD4+ CCR7+ CD45RA+) [41]. Another viral infection in which IL-35 is implicated is influenza A. It has been shown that, during influenza A infection, IL-35 concentrations are increased in human lung and epithelial samples, as well as peripheral blood mononuclear cells, through the activation of the NF-_Κ_B pathway [37,42]. These results imply that IL-35 inhibits the early immune response and, while this might be beneficial in some cases such as allergy and hyperinflammatory events, it could contribute to secondary pneumococcal pneumonia susceptibility during influenza A infection [42].

Studies have shown that IL-35 correlates positively with CD4+ T-cells and Tregs, and negatively with CD8+ cytotoxic T-cells [43]. Similar findings were observed in the present study, especially in PLWH, showing a large negative correlation between IL-35 and CD8+ EM2 cell percentages. Whether IL-35 is induced by the host to prevent inflammation-mediated tissue damage or by the pathogen to facilitate survival and replication is unknown [43]. Regardless of the mechanism, higher IL-35 levels may protect against severe inflammation during HIV- and SARS-CoV-2 co-infection.

CD8+ EM4 T-cells are functionally similar to CD8+ CM cells, expressing low levels of effector molecules like perforin and granzyme B [44]. CD8+ CD57+ populations emerge in infections with repeated antigen stimulation, such as HIV [44]. CD8+ T-cell exhaustion might be clinically beneficial during acute COVID-19 by limiting tissue damage from SARS-CoV-2-specific CD8+ T-cells [45]. However, a loss of CD8+ T-cell function, as seen in exhausted and senescent T-cells, could hinder viral clearance, leading to persistent SARS-CoV-2 infection, as shown by Karim et al. [25,46]. These authors studied the T-cell responses in PLWH with advanced disease, finding prolonged SARS-CoV-2 infection due to T-cell depletion, a rare occurrence in patients with competent T-cell responses [46]. The continuous over-expression of the inhibition marker PD-1 on CD8+ T-cells reduces TNF-α and IFN-γ production [9]. In our study, PLWH were found to have higher percentages of CD8+ EM4 T-cells expressing both PD-1 and CD57 at V2. In support of this contention, Petrovas et al. reported that the co-expression of CD57 and PD-1 indicates a T-cell population more prone to apoptosis, which is more frequent in PLWH [47].

Diverse immune responses are observed in PLWH owing to their varying CD4+ T-cell counts and HIV VL. Previous studies have shown poor outcomes in PLWH with CD4+ T-cell counts of <200 cells/mm^3^ at hospital admission [11,19,48,49]. In the current study, PLWH with CD4+ T-cell counts of <200 cells/mm^3^ exhibited lower IL-29 and higher IL-6 levels. IL-29, which is produced by macrophages and DCs, promotes antiviral activity and the differentiation of FOXP3-expressing suppressor T-cells [50]. The innate immune system, particularly type I interferons (T1IFNs), plays a crucial role in antiviral defense. SARS-CoV-2, like other coronaviruses, can delay T1IFN production and inhibit T1IFN signaling, weakening early immune responses [50]. Type I and III interferons are essential in defending the host against viruses, and SARS-CoV-2 is sensitive to pretreatment with these IFNs in vitro [50]. IL-29, a type III interferon, shows antiviral activity similar to T1IFNs and has been found to decrease the disease severity and transmission of SARS-CoV-2 in animal models [50]. Vastani et al. found that COVID-19-related ARDS survivors had higher IL-29 levels than non-survivors, suggesting a protective role in SARS-CoV-2 infection and its potential as a predictor of severe disease [50]. IL-29 stimulates immune-regulating functions and may help to improve the condition of patients with COVID-19 [50]. Lower levels of IL-29 have been proposed to predict severe COVID-19, and higher IL-29 levels correlate with better immune cell counts [50].

Lower IL-29 levels in PLWH with CD4+ T-cell counts of <200 cells/mm^3^ may hinder Treg development, which is crucial for controlling exaggerated CD8+ T-cell responses. SARS-CoV-2 induces innate immune cells to produce cytokines like IL-6 [51]. While IL-6 is vital for viral infection control, its overproduction can lead to hypercytokinemia, increased vascular permeability, and respiratory and multi-organ failure [52]. High IL-6 levels have been linked to severe COVID-19 and mortality, and, accordingly, has been proposed as a marker of disease progression [51,52,53]. While IL-6 receptor blocking therapy has become the mainstay of immunomodulatory treatment for COVID-19 in high-income countries, it might not be a suitable option for PLWH. A case series study performed in the United States of America investigating whether IL-6 inhibitors could be beneficial as a treatment option for PLWH admitted to the hospital with COVID-19 found that there were multiple reports of secondary infections [54]. This study included 18 PLWH, of whom 4 (22%) had CD4+ T-cell counts of <200 cells/mm^3^ [54]. The same was true for a clinical trial carried out in sub-Saharan Africa [55]. The study tested the efficacy of tocilizumab in patients with acute COVID-19, of which 2.3% (21/913) were PLWH [55]. Patients treated with tocilizumab had higher rates of secondary infections compared to those not receiving the drug (17.2% vs. 4.8%, *p* < 0.001) [55]. The study further found no significant improvement in the mortality rate of patients with COVID-19 receiving tocilizumab [55]. This would indicate that it might not be the most effective treatment for PLWH due to their already weakened immune response. Clearly, more studies are needed.

PLWH with a detectable VL exhibited significantly lower CD4+ T-cell percentages and higher CD8+ T-cell percentages, as well as higher percentages of CD4+ and CD8+ EM subsets and CD8+ T-cells expressing PD-1, which is typical of PLWH with viremia [56]. Their cytokine profile suggested a reduced ability to respond to SARS-CoV-2, with lower levels of IL-2, IL-4, IL-12p40, and IFN-γ, which are crucial for T-cell function and infection control. IL-2 is a pleiotropic cytokine involved in T-cell survival, differentiation, and proliferation. Lower numbers of pre-existing naïve CD4+ T-cells, in combination with the overexpression of memory CD8+ T-cells and less production of IL-2 and IFN-γ in the context of HIV, could lead to decreased priming and dysregulated early and subsequent memory immune responses to SARS-CoV-2.

HIV proteins can block the production of IL-12 by monocytic lineages [57]. In this context, a recent study showed that IL-12p40 can reduce autoimmune signaling through the inhibition of IL-12Rβ1 internalization, indicating a possible role for this cytokine in the anti-inflammatory response [58]. Another study by Marks et al. demonstrated that IL-12p40 can be selectively regulated by hypoxia inducible factor (HIF), which is expressed during hypoxia, and has been shown to be upregulated during severe SARS-CoV-2 infection [59]. The study proposed that the HIF-IL-12p40 axis may be a protective mechanism to limit immune cell influx into inflamed tissue, thus regulating inflammation by switching the production of pro-inflammatory IL-12p70 to an antagonistic IL-12p40 [59]. This aligns with Nkosi et al. and Chanda et al., who found diminished cellular responses and lower frequencies of SARS-CoV-2-specific IFN-γ-producing CD4+ T-cells in PLWH with a detectable HIV VL, leading to weakened immune responses to SARS-CoV-2 and, thus, a higher susceptibility to severe disease, poorer COVID-19 outcomes, and mortality [23,48]. A multicenter study carried out with data from 54 clinical sites in the USA, including 13,170 PLWH, reported that no association could be found between HIV viral suppression and COVID-19 severity or mortality [49].

In the current study, we found that PLWH who were hospitalized with COVID-19 that were not undergoing ART had lower concentrations of IL-2, IL-10, and TGF- β, but no difference could be found in terms of IFN-γ. A study by Sharov et al. found that HIV and COVID-19 together exacerbate immune system degradation, with PLWH not undergoing ART having lower serum concentrations of IL-2, IFN-γ, and TNF-α, which they concluded indicates a weakened immune response and can exacerbate COVID-19 symptoms, leading to more severe disease in PLWH not undergoing ART [60]. In contrast, in their study and ours, PLWH undergoing ART showed a more effective immune response and fewer complications [60]. This underscores the importance of ART in managing co-infections and preventing severe immunological deterioration in PLWH.

A major limitation of this study is that the link between IL-35 and Treg involvement is, at this point, speculative, and should be confirmed by further studies such as the phenotyping of Tregs, as well as in vitro studies, both of which will be necessary to make this statement more definitive. Another limitation is that, during the pandemic, due to the burden of a large influx of COVID-19 admissions and short-staffed hospital environments, some clinical records were incomplete at V2, and, thus, a full analysis comparing the disease progression between the groups could not be performed. We also acknowledge that our relatively small sample size limits the power of this study and that a potential recruitment bias in favor of PLWH could have influenced the participant disease profiles. It is also possible that the immune responses measured could have been influenced by undisclosed treatments administered before hospitalization.

## 5. Conclusions

Differences in the T-cell phenotypes and cytokine profiles between people with and without HIV admitted to hospitals with COVID-19 were explored. Despite PLWH being associated with chronic systemic inflammation, in the context of co-infection with SARS-CoV-2, PLWH had lower percentages of CD8+ EM T-cells compared to people living without HIV after ±7 days since admission to a hospital. This finding may be indicative of suppressive Treg mechanisms inhibiting EM T-cell survival, as indicated by the higher expression of IL-35 and the T-cell maturation arrest observed in PLWH. When compared to people without HIV, PLWH with CD4+ T-cell counts of ≥200 cells/mm^3^, indicative of partial immune competence, had altered CD4+ and CD8+ T-cell profiles, in the setting of lower levels of systemic inflammation, as measured by plasma ferritin and PCT levels, and less severe disease, as indicated by a decreased demand for oxygen. On the other hand, this profile was not seen in PLWH with severe immunodeficiency, highlighting the need for differentiated care in the broader PLWH population. This also raises the question of the potential value of adjuvant immunotherapeutic strategies in the setting of severely compromised CD4+ T-cell counts.

## Figures and Tables

**Table 1:** Demographic and clinical characteristics of people living with and without HIV at admission to hospital with COVID-19.

	COVID-19+ PLWOH (*n* = 137)	COVID-19+ PLWH (*n* = 37)	*p*-Value
Age (years)	54 ± 14	46.5 ± 11	**0.001**
Sex (male)	81/137 (59%)	11/37 (30%)	**0.002**
Diabetes	48/122 (39%)	6/37 (19%)	**0.003**
MTB: Current	1/122 (1%)	3/36 (8%)	**0.011**
MTB: Past	2/122 (2%)	5/36 (14%)	**0.002**
FiO_2_ Normal range: 0.21	0.53 (0.21–0.90)	0.26 (0.21–0.44)	**0.014**
PaO_2_ (mmHg) Normal range: 75–100	52.4 (31.8–69.1)	55.8 (48.2–71.5)	0.337
P/F ratio Normal range: ≥400	101.96 (70–155.9)	128.75 (107.6–306.7)	**0.021**
Ferritin (μg/L) Normal range: 5–204	748 (325–1591)	269.5 (79–855)	**0.002**
CRP (mg/L) Normal range: <10	106 (58–195)	116 (57–189)	0.734
PCT (μg/L) Normal range: <0.05	0.13 (0.06–0.34)	0.05 (0.03–0.15)	**0.047**
Outcome (Deceased)	22/134 (16.4%)	4/37 (10.8%)	0.668
ART		26/37 (70.3%)	
HIV VL (copies/mL)		20 (20–12,980)	
CD4+ T-cell count (cells/mm^3^)		256 (115–388)	

Abbreviations: Antiretroviral therapy (ART), cluster of differentiation (CD), C-reactive protein (CRP), fraction of inspired oxygen (FiO_2_), human immunodeficiency virus (HIV), *Mycobacterium tuberculosis* (MTB), Horowitz Index for Lung Function (P/F ratio), procalcitonin (PCT), people living with HIV (PLWH), people living without HIV (PLOWH), and viral load (VL). Significant *p*-values are indicated in bold.

**Table 2:** Stepwise backward logistic multivariable regression model of T-cell populations associated with PLWH hospitalized with COVID-19 at Visit 1.

HIV	Odds Ratio	Std. Err.	z	*p* > z	[95% Conf. Interval]	
Age	0.913	0.034	−2.43	**0.015**	0.848	0.983
CD4+ CM PD-1+	1.170	0.047	3.87	**<0.001**	1.081	1.267
Sex	0.220	0.176	−1.89	0.059	0.046	0.983
CD8+ N	1.053	0.030	1.80	0.072	0.996	1.113
CD8+ EM3	1.053	0.029	1.87	0.061	0.998	1.111
CD8+ EM2	1.284	0.084	3.81	**<0.001**	1.129	1.461
CD8+ EM4 CD57+	3.827	1.781	2.88	**0.004**	1.537	9.530
IL-19	0.913	0.008	−2.77	**0.006**	0.961	0.993
IL-35	1.028	0.008	3.55	**<0.001**	1.012	1.043
Constant	3.84 × 10^−7^	1.66 × 10^−6^	−3.42	**0.001**	8.19 × 10^−11^	0.002

Abbreviations: central memory (CM), cluster of differentiation (CD), double positive (DP), effector memory (EM), naïve (N), interleukin (IL), and programmed cell death protein 1 (PD-1). Model characteristics: *n* = 137, LR chi2(9) = 104.70, Prob < 0.001, Pseudo R2(0.672), Log likelihood = −25.50, goodness-of-fit = 0.391, 93.43% correctly classified. Significant *p*-values are indicated in bold.

**Table 3:** Logistic regression model by HIV status at Visit 2.

HIV	Odds Ratio	Std. Err.	z	*p* > z	(95% Conf. Interval)	
Age	0.955	0.071	−0.62	0.538	0.826	1.105
CD4+ CM PD-1+	1.408	0.223	2.16	**0.031**	1.032	1.920
CD8+ EM4 CD57+ PD-1+	20.841	30.991	2.04	**0.041**	1.130	384.299
CD8+ TEMRA	0.793	0.098	−1.88	0.060	0.622	1.010
CD8+ EM	0.814	0.072	−2.33	**0.020**	0.684	0.968
CD8+	1.402	0.190	2.49	**0.013**	1.084	1.829
constant	<0.001	<0.001	1.51	0.130	0.005	1.23 × 10^18^

Abbreviations: central memory (CM), effector memory (EM), programmed cell death protein 1 (PD-1), and terminally differentiated T-cells re-expressing CD45RA. *n* = 69, LR chi2(5) = 56.51, Prob < 0.001, Pseudo R2(0.734). Significant *p*-values are indicated in bold.

## Data Availability

The raw data supporting the conclusions of this article will be made available by the authors on request.

## Supplementary Materials

The following supporting information can be downloaded at: https://www.mdpi.com/article/10.3390/microorganisms12112149/s1.
