# Supplementary material for: T-Cell Phenotypes and Systemic Cytokine Profiles of People Living with HIV Admitted to Hospital with COVID-19
Source: Microorganisms. 2024 Oct 25;12(11):2149. doi: 10.3390/microorganisms12112149 (PMC11596914; doi:10.3390/microorganisms12112149)
Supplement: Supplementary file 1 [file microorganisms-12-02149-s001.zip › microorganisms-3211613-supplementary.pdf]

Supplementary material

T-Cell Phenotypes and Systemic Cytokine Profiles of People Living with HIV Admitted to Hospital with COVID-19

Mieke A. van der Mescht [1], Helen C. Steel [1], Zelda de Beer [1,2], Andries Masenge [3], Fareed Abdullah [4,5,6], Veronica Ueckermann [4], Ronald Anderson [1] and Theresa M. Rossouw [1,*]

[1] Department of Immunology, School of Medicine, Faculty of Health Sciences, University of Pretoria, Pretoria 0001, South Africa; u14033692@tuks.co.za (M.A.v.d.M.); helen.steel@up.ac.za (H.C.S.); zeldavdwalt22@gmail.com (Z.d.B.); ronald.anderson@up.ac.za (R.A.)

[2] Tshwane District Hospital, Pretoria 0084, South Africa

[3] Department of Statistics, Faculty of Natural and Agricultural Sciences, University of Pretoria, Pretoria 0001, South Africa; andries.masenge@up.ac.za

[4] Division for Infectious Diseases, Department of Internal Medicine, Steve Biko Academic Hospital, University of Pretoria, Pretoria 0001, South Africa; fareed.abdullah@mrc.ac.za (F.A.); veronica.ueckermann@up.ac.za (V.U.)

[5] Office of AIDS and TB Research, South African Medical Research Council, Pretoria 0001, South Africa

[6] Department of Public Health Medicine, Faculty of Health Sciences, University of Pretoria, Pretoria 0001, South Africa

* Correspondence: theresa.rossouw@up.ac.za

Keywords: SARS-CoV-2; COVID-19; HIV; T-cells; cytokines

Supplementary Table 1: Univariate comparison of T-cell profiles of people with and without HIV at admission with COVID-19, as well as controls

T-cell population	COVID+PLWOH (n=117) Group 1	Control PLWOH (n=10) Group 2	COVID+PLWH (n=36) Group 3	Control PLWH (n=19) Group 4	1 vs 2	1 vs 3	3 vs 4	2 vs 4	Overall p-value
CD4+	66.51 (54.66 - 76.47)	73.00 (65.88-79.63)	48.96 (17.23 - 58.12)	42.56 (22.23-54.93)	0.110	**<0.001**	0.336	**<0.001**	**<0.001**
CD8+	27.77 (18.91-37.08)	22.92 (15.18-28.04)	47.86 (36.02-73.5)	53.13 (39.30-70.11)	0.151	**<0.001**	0.404	**<0.001**	**<0.001**
DP	0.84 (0.47-1.73)	0.50 (0.40-0.92)	0.44 (0.23-0.76)	0.55 (0.19-0.81)	0.132	**<0.001**	0.461	0.195	**<0.001**
DN	3.28 (2.19-4.78)	3.70 (2.74-5.39)	4.58 (2.48-6.28)	5.47 (3.66-7.44)	0.223	**0.015**	0.083	0.076	**0.004**
CD8+ PD-1+	35.09 (21.11-44.27)	27.66 (19.59-36.61)	37.76 (27.89-51.04)	34.96 (30.86-48.30)	0.197	**0.032**	0.375	0.082	0.146
CD4+ CM	53.45 (44.96-65.76)	53.81 (44.70-58.86)	50.66 (37.52-58.72)	51.96 (44.45-59.22)	0.470	**0.025**	0.132	0.221	0.252
CD4+ EM	10.97 (6.93-17.2)	7.76 (5.46-9.35)	17.88 (9.61-31.90)	23.36 (13.96-31.79)	0.071	**0.002**	0.109	**<0.001**	**<0.001**
CD4+ EM2	0.51 (0.22-0.74)	0.31 (0.12-0.60)	0.34 (0-0.54)	0.82 (0.27-1.81)	0.212	**0.018**	**0.003**	0.050	**0.033**
CD4+ CM PD-1+	37.66 (29.59-47.76)	33.72 (24.64-37.91)	57.86 (51.18-73.30)	53.00 (45.61-73.03)	0.051	**<0.001**	0.229	**<0.001**	**<0.001**
CD8+ EM1	46.64 (27.69-60.5)	64.74 (59.49-70.49)	32.66 (24.06-42.73)	38.64 (19.21-46.76)	**0.010**	**<0.001**	0.268	**0.001**	**0.002**
CD8+ EM2	13.42 (9.63-20.24)	11.55 (7.28-14.80)	23.94 (15.35-35.40)	18.13 (8.69-43.22)	0.131	**<0.001**	0.090	**0.016**	**<0.001**
CD8+ EM1 PD-1+	35.97 (21.72-49.78)	40.86 (37.80-59.26)	25.37 (21.10-35.43)	19.10 (10.99-35.10)	0.085	**0.012**	0.131	**0.001**	**0.001**
CD8+ EM2 PD-1+	9.72 (4.84-14.42)	6.72 (4.51-9.93)	18.3 (12.24-29.91)	11.00 (3.41-33.25)	0.113	**<0.001**	**0.006**	0.052	**<0.001**

CD8+ EM3 PD-1+	9.18 (4.45-21.02)	5.55 (2.66-11.13)	13.89 (9.49-24.84)	17.82 (6.55-21.28)	0.0713	**0.008**	0.317	**0.020**	0.073
CD8+ TEMRA ECD57+	52.4 (37.11-62.61)	40.29 (21.49-53.80)	44.07 (25.91-52.95)	50.95 (31.87-61.15)	**0.035**	**0.006**	0.146	0.143	**0.036**
CD8+ TEMRA pE1	5.43 (3.04-10.21)	12.14 (4.42-17.38)	4.37 (2.62-7.01)	5.55 (2.83-13.12)	0.062	**0.027**	0.115	0.086	0.071
CD8+ CM PD-1+	45.64 (36.05-56.02)	51.26 (42.11-54.99)	62.22 (55.68-68.67)	55.34 (44.71-65.50)	0.325	**<0.001**	0.069	0.109	**<0.001**

The Kruskal-Wallis test with post hoc Dunn test was used to compare continuous variables between groups. Results are presented as median and interquartile range (IQR).

Abbreviations: Central memory (CM), double -negative (DN), double -positive (DP), end stage effector (E), effector memory (EM), people living without HIV (PLWOH), people living with HIV (PLWH), programmed cell death protein 1 (PD-1), pre-effector 1 (pE1), terminally differentiated T-cells re-expressing CD45RA (TEMRA).

Supplementary Table S2: Univariate comparison of T-regulatory cell cytokines of people with and without HIV at admission with COVID-19, as well as controls.

**Treg marker (pg/mL)**	**COVID+PLWOH (n=102) Group 1**	**Control PLWOH (n=8) Group 2**	**COVID+PLWH (n=35) Group 3**	**Control PLWH (n=8) Group 4**	**1 vs 2**	**1 vs 3**	**3 vs 4**	**2 vs 4**	**Overall p-value**
IL-2	6.33 (2.48-19.12)	0.25 (0.25-2.67)	7.88 (2.48-15.52)	0.26 (0.25-8.26)	**0.003**	0.489	**0.025**	**0.307**	**0.008**
IL-10	3.37 (1.11-3.37)	1.11 (1.11-1.11)	3.37 (1.11-3.37)	1.11 (1.11-1.11)	**0.011**	0.368	**0.009**	**0.492**	**0.013**
IL-12p40	125.45 (87.66-180.04)	144.33 (57.21-134.65)	129.14 (77.92-178.12)	112.48 (82.38-136.36)	0.164	0.343	0.298	0.443	0.682
IL-19	72.54 (13.22-132.94)	3.78 (3.78-3.79)	75.87 (13.22-104.94)	38.17 (3.78-132.35)	**<0.001**	0.386	0.196	**0.055**	**0.010**
IL-20	35.52 (15.92-63.05)	22.37 (4.24-28.76)	41.37 (6.91-56.9)	17.54 (4.24-38.22)	**0.027**	0.483	**0.041**	0.484	0.073
IL-22	2.69 (2.02-2.69)	2.69 (2.69-2.69)	2.69 (2.02-2.69)	2.69 (2.69-2.69)	0.189	0.384	0.165	0.500	0.631
IL-26	358.6 (100.27-358.6)	100.27 (100.27-100.27)	358.6 (100.27-358.66)	100.27 (100.27-100.27)	**0.004**	0.392	**<0.001**	**0.245**	**<0.001**
IL-28A	26.56 (13.91-48.3)	9.12 (2.17-12.93)	28.81 (5.82-49.74)	13.69 (10.65-31.07)	**0.004**	0.488	0.114	**0.162**	**0.037**
IL-29	29.64 (10.85-52.67)	46.36 (11.94-57.74)	33.08 (6.99-52.67)	19 (7.89-38.71)	0.234	0.464	0.270	0.164	0.809
IL-35	97.6 (13.78-180.85)	126.10 (43.84-188.46)	116.51 (50.82-199.85)	95.2 (31.15-162.48)	0.296	0.157	0.328	0.364	0.757
IL-27p28	16.01 (5.05-16.01)	5.05 (5.05-18.98)	16.01 (16.01-16.01)	5.05 (5.05-8.48)	0.062	0.072	**0.002**	**0.303**	**0.013**

The Kruskal-Wallis test with post hoc Dunn's test was used to compare continuous variables between groups. All variables are shown as median (interquartile range) in picogram (pg)/mL.

Abbreviations: people living without HIV (PLWOH), interleukin (IL), people living with HIV (PLWH), T-regulatory cell (Treg)

Supplementary Table S3: Univariate comparison of pro- and anti-inflammatory cytokines of people with and without HIV at admission with COVID-19, as well as controls.

Cytokine	COVID+PLWOH (n=137) Group 1	Control PLWOH (n=9) Group 2	COVID+PLWH (n=37) Group 3	Control PLWH (n=9) Group 4	1 vs 2	1 vs 3	3 vs 4	2 vs 4	Overall p-value
TGF-β1 (ng/mL)	8.08 (5.73-11.85)	5.02 (3.10-5.73)	7.78 (5.20-11.57)	3.24 (3.16-7.12)	**0.007**	0.479	**0.005**	**0.020**	**0.004**
IL-1β (pg/mL)	1.95 (1.46-2.89)	1.22 (0.98-1.71)	1.95 (1.46-2.66)	0.72 (0.59-0.72)	**0.009**	0.406	**<0.001**	**0.039**	**<0.001**
IL-1Ra (pg/mL)	812.8 (631.27-1081.29)	237.73 (214.08-407.80)	894.53 (725.59-1133.51)	259.97 (214.08-554.28)	**<0.001**	0.104	**<0.001**	**0.010**	**<0.001**
IL-4 (pg/mL)	4.14 (3.07-5.49)	2.83 (2.07-3.79)	4.54 (3.9-5.49)	2.7 (2.32-3.19)	**0.016**	0.105	**<0.001**	0.080	**0.002**
IL-6 (pg/mL)	4.09 (1.54-8.93)	0.49 (0.30-0.54)	6.38 (2.29-13.18)	0.49 (0.49-0.77)	**<0.001**	0.102	**<0.001**	**0.010**	**<0.001**
IL-7 (pg/mL)	45.82 (28.96-64.84)	10.79 (6.88-27.05)	41.79 (71.89-27.05)	17.74 (10.79-38.34)	**<0.001**	0.445	**0.004**	0.128	**<0.001**
IL-8 (pg/mL)	17.5 (10.73-23.53)	3.58 (2.8-6.47)	19.69 (15.32-28.23)	4.62 (3.84-6.47)	**<0.001**	0.114	**<0.001**	**0.011**	**<0.001**
IL-13 (pg/mL)	3.61 (2.18-5.4)	1.43 (0.91-1.55)	3.2 (1.76-5.01)	0.91 (0.47-1.76)	**<0.001**	0.267	**<0.001**	0.054	**<0.001**
IL-15 (pg/mL)	18.02 (18.02-18.02)	18.02 (18.02-18.02)	18.02 (18.02-18.02)	18.02 (18.02-18.02)	0.227	**0.032**	0.247	0.268	0.226
IL-17 (pg/mL)	22.61 (17.36-32.32)	12.58 (10.37-19.62)	24.85 (18.74-34.51)	15.22 (12.14-19.62)	**0.009**	0.223	**0.007**	0.172	**0.008**
Eotaxin (pg/mL)	22.81 (15.84-31.11)	33.93 (26.19-42.79)	27.01 (20.02-35.66)	32.93 (23.68-33.75)	**0.010**	**0.025**	0.414	0.334	**0.027**
IFN-γ (pg/mL)	15.81	6.75	16.93	4.99	**<0.001**	0.186	**<0.001**	0.090	**<0.001**

	(10.7-23.01)	(4.54-10.67)	(12.37-26.35)	(4.1-9.37)					
IP-10 (pg/mL)	952.33 (421.09-2087.96)	160.02 (129.86-208.83)	680.22 (415.47-1442.47)	261.01 (213.06-763.50)	**<0.001**	0.207	**0.024**	0.308	**<0.001**
TNF-α (pg/mL)	83.87 (68.6-102.39)	55.49 (50.67-68.60)	90.85 (73.41-118.4)	50.67 (47.67-81.53)	**<0.001**	0.100	**0.005**	0.403	**<0.001**

The Kruskal-Wallis test with post hoc Dunn’s test was used to compare continuous variables between groups. All variables are shown as median (interquartile range) in picograms (pg)/mL with the exception of TGF-β1which is expressed in nanograms (ng)/mL.

Abbreviations: people living without HIV (PLWOH), interleukin (IL), IL-1 receptor antagonist (IL-1Ra), interferon gamma (IFN-γ), interferon gamma-induced protein 10 (IP-10), people living with HIV (PLWH), transforming growth factor-β1 (TGF-β1), tumor necrosis factor-α (TNF-α).

Supplementary Table S4: Univariate comparison of the cytokine profiles of people living with HIV according to CD4 count at admission with COVID-19, as well as with their respective controls.

**Cytokine**	**COVID CD4 ≥200 (n=19)** **Group 1**	**Control CD4 ≥200 (n=4)** **Group 2**	**COVID CD4 <200 (n=17)** **Group 3**	**Control CD4 <200 (n=5)** **Group 4**	**1 vs 2**	**1 vs 3**	**3 vs 4**	**2 vs 4**	**Overall p-value**
TGF-β1 (ng/mL)	7.98 (5.20-17.06)	3.91 (3.16-8.42)	7.35 (5.08-11.57)	3.24 (2.70-7.12)	**0.046**	0.289	**0.032**	0.380	0.074
IL-1β (pg/mL)	1.95 (1.35-3.69)	0.66 (0.39-0.85)	2.03 (1.71-2.43)	0.72 (0.59-0.72)	**0.002**	0.443	**0.022**	0.227	**0.007**
IL-1Ra (pg/mL)	894.53 (655.64-1133.51)	237.03 (153.06-407.13)	952.91 (747.98-1175.93)	301.18 (214.08-580.65)	**0.005**	0.298	**0.004**	0.360	**0.003**
IL-2 (pg/mL)	9 (4.23-10.99)	1.63 (1.26-1.63)	5.47 (1.63-10.30)	1.63 (1.63-1.71)	**0.001**	0.141	0.077	0.191	**0.008**
IL-4 (pg/mL)	4.54 (3.9-6.47)	2.95 (2.39-3.31)	4.70 (3.98-5.04)	2.45 (2.32-2.70)	**0.011**	0.430	**0.035**	0.343	**0.037**
IL-6 (pg/mL)	4.32 (1.25-7.28)	0.49 (0.28-0.63)	13.18 (5.39-72.30)	0.49 (0.49-1.01)	**0.029**	**0.009**	**0.001**	0.343	**0.001**
IL-7 (pg/mL)	50.12 (35.61-96.45)	16.06 (12.59-28.04)	35.53 (24.05-58.82)	17.74 (4.7-50.12)	**0.004**	0.059	0.136	0.278	**0.017**
IL-8 (pg/mL)	18.59 (14.45-20.24)	5.15 (4.1-6.21)	23.81 (15.32-31.61)	4.62 (1.78-7.53)	**0.015**	0.091	**0.001**	0.465	**0.002**
IL-13 (pg/mL)	3.61 (2.59-7.56)	0.80 (0.47-1.39)	3.05 (1.55-4.61)	0.91 (0.47-1.76)	**0.004**	0.122	**0.026**	0.439	**0.006**
IL-15 (pg/mL)	18.02 (18.02-109.12)	18.02 (18.02-18.02)	18.02 (18.02-18.02)	18.02 (18.02-18.02)	0.106	0.124	0.353	0.232	0.521
IL-17 (pg/mL)	26.64 (16.1-35.38)	15.88 (9.26-20.50)	23.36 (18.74-34.51)	15.22 (13.46-18.74)	**0.021**	0.361	0.097	0.308	0.119
Eotaxin (pg/mL)	26.38	33.34	30.25	25.01	0.125	0.188	0.217	0.137	0.566

	(19.45-33.39)	(28.31-39.61)	(21.89-37.84)	(15.05-33.57)					
IFN-γ (pg/mL)	16.23 (8.66-29.31)	4.50 (1.37-12.15)	20.48 (14.53-26.35)	4.99 (4.54-9.37)	**0.026**	0.134	**0.007**	0.389	**0.014**
IP-10 (pg/mL)	554.9 (338.98-981.23)	349.50 (181.59-629.52)	854.15 (672.38-1701.47)	261.01 (252.58-922.75)	0.099	**0.035**	**0.034**	0.285	**0.046**
TNF-α (pg/mL)	84.60 (73.41-124.78)	52.46 (47.03-69.71)	93.16 (76.85-116.12)	50.67 (48.25-114.98)	**0.019**	0.378	0.115	0.174	0.111

The Kruskal-Wallis test with post hoc Dunn's test was used to compare continuous variables between groups. All variables are shown as median (interquartile range) in picograms (pg)/mL with the exception of TGF-β1which is expressed in nanograms (ng)/mL.

Abbreviations: Interleukin (IL), IL-1 receptor antagonist (IL-1Ra), interferon gamma (IFN-γ), interferon gamma-induced protein 10 (IP-10), transforming growth factor-β1 (TGF-β1), tumor necrosis factor-α (TNF-α).

Supplementary Table S5: Univariate comparison of the regulatory cytokine profiles of people living with HIV according to CD4 count at admission with COVID-19, as well as with their respective controls.

Treg marker (pg/mL)	COVID CD4 ≥200 (n=19) Group 1	Control CD4 ≥200 (n=4) Group 2	COVID CD4<200 (n=17) Group 3	Control CD4<200 (n=5) Group 4	1 vs 2	1 vs 3	3 vs 4	2 vs 4	p-value
IL-2	6.99 (2.48-24.14)	6.47 (0.25-13.77)	7.88 (2.48-12.85)	0.26 (0.25-2.05)	0.207	0.421	**0.033**	0.180	0.217
IL-10	3.37 (1.11-3.37)	1.11 (1.11-2.58)	3.37 (2.24-5.52)	1.11 (1.11-1.11)	0.191	0.118	**0.005**	0.215	**0.048**
IL-12p40	143.81 (87.66-182.42)	136.36 (97.41-172.80)	91.86 (52.33-156.37)	97.45 (68.83-112.48)	0.489	0.084	0.318	0.143	0.367
IL-19	85.32 (13.22-104.94)	59.21 (3.78-136.46)	80.20 (13.22-109.27)	38.17 (3.79-111.30)	0.418	0.446	0.262	0.392	0.930
IL-20	42.93 (22.38-63.63)	30.21 (10.95-49.15)	39.46 (6.91-53.40)	10.84 (2.51-25.55)	0.219	0.187	**0.048**	0.127	0.161
IL-22	2.69 (2.02-32.3)	2.69 (2.69-2.69)	2.03 (2.02-2.69)	2.69 (2.69-2.69)	0.360	0.071	0.105	0.500	0.337
IL-26	358.60 (100.27-358.60)	100.27 (100.27-100.27)	358.60 (229.44-358.60)	100.27 (100.27-100.27)	**0.011**	0.323	**0.005**	0.500	**0.008**
IL-28A	33.32 (15.21-53.55)	19.74 (12.17-31.07)	27.12 (4.16-43.85)	12.17 (6.04-25.78)	0.226	0.251	0.222	0.365	0.623
IL-29	50.19 (27.02-57.47)	28.09 (7.89-58.85)	19.00 (7.00-33.08)	19.00 (7.89-29.62)	0.275	**0.016**	0.426	0.235	0.140
IL-35	124.21 (50.82-199.85)	126.97 (43.84-196.55)	106.35 (56.28-174.00)	82.51 (31.14-130.71)	0.482	0.348	0.226	0.227	0.790
IL-27p28	16.01 (16.01-26.98)	8.48 (5.05-34.40)	16.01 (16.01-16.01)	5.05 (5.05-5.05)	0.100	0.351	**0.005**	0.113	**0.028**

The Kruskal-Wallis test with post hoc Dunn's test was used to compare continuous variables between groups. All variables are shown as median (interquartile range) in picograms (pg)/mL.

Abbreviations: Interleukin (IL), T-regulatory cell (Treg)

Differences in the T-cell profiles of people with COVID-19 without HIV between V1 and V2

Differences were found between bloods taken at visit 1 (V1) and visit 2 (V2) during hospitalization for COVID-19 (Supplementary Table S6 and Figure S1). At the V2 timepoint, the percentages of CD4+ T-cells increased (p=0.019) and CD8+ T-cells decreased (p=0.025). The increase was seen within the CD8+ effector memory 2 (EM2: CCR7-CD45RA-CD28-CD27+) (p=0.001) subset, specifically CD8+ EM2 T-cells expressing PD-1 (p<0.001). A difference was also found between V1 and V2 for the TEMRA subset. There was an increase in the percentage of CD8+ end-stage effector (E: CCR7-CD45RA+CD28-CD27-) T-cells co-expressing CD57 and PD-1 (p=0.019).

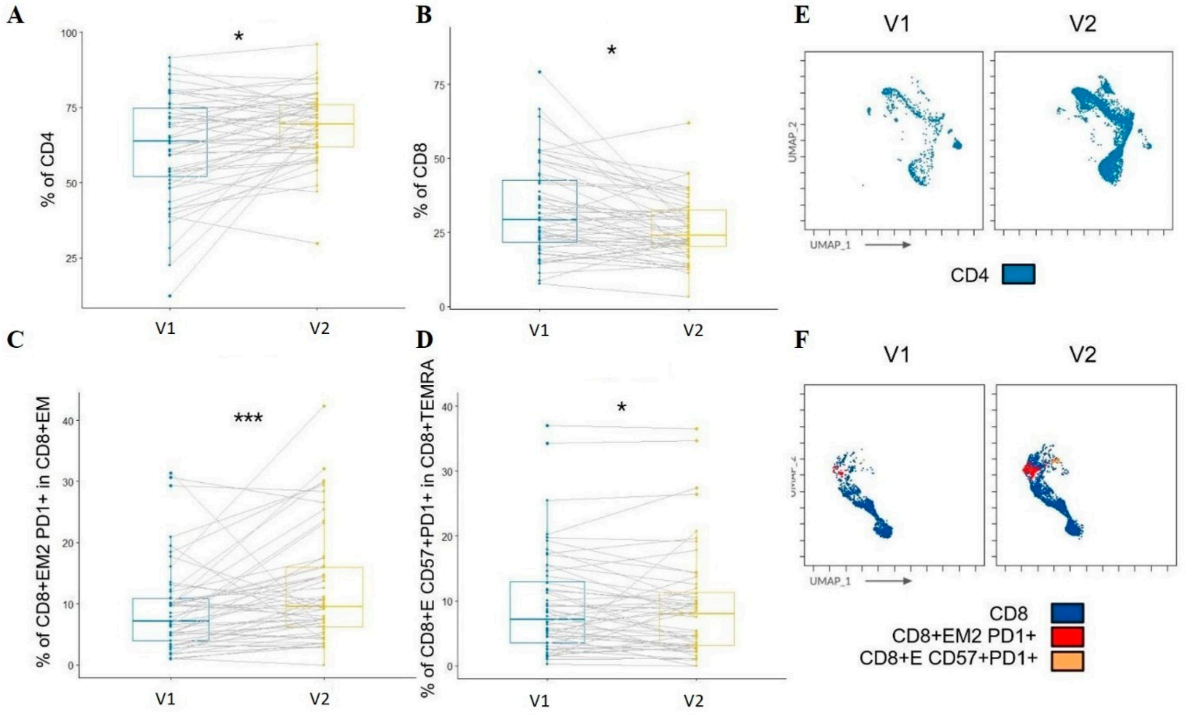

Supplementary Figure S1: Differences in the T-cell phenotypes of people with COVID-19 without HIV at hospital admission (V1) and 5 ± 3.6 days after admission (V2). **A**: The percentage of CD4+ T-cells increased from V1 to V2. **B**: The percentage of CD8+ T-cells decreased from V1 to V2. **C**: The percentage of CD8+ EM2 expressing PD-1 increased from V1 to V2. **D**: Boxplots show an increase in the percentage of CD8+ E co-expressing CD57 and PD-1 from V1 to V2. **E**: A representative UMAPs showing an increase of CD4+ T-cells between V1 and V2 in people without HIV admitted with COVID-19. **F**: Representative UMAP plots show the increases/decreases in the respective CD8+ T-cell populations between V1 and V2 in people without HIV admitted with COVID-19.

p-value: * = < 0.05, ** = <0.01.

Abbreviations: End-stage effectors (E), effector memory (EM), programmed cell death protein 1 (PD-1), visit 1 (V1), visit 2 (V2)

Supplementary Table S6: Differences in T-cell populations between V1 and V2 in people without HIV hospitalized with COVID-19.

T-cell population	COVID-19+ PLWOH V1 (n=52)	COVID-19+PLWOH V2 (n=52)	p-value
CD4+	63.85 (51.74-74.73)	69.59 (61.93-75.98)	**0.019**
CD8+	29.32 (21.27-43.22)	24.02 (19.92-32.93)	**0.025**
CD4+ EM4 PD-1+	2.41 (1.23-4.90)	4.10 (2.16-5.55)	0.080

CD8+ EM2 CD57+PD-1+	3.27 (1.50-6.65)	4.47 (2.37-7.57)	**0.019**
CD8+ EM4 CD57+ PD-1+	0.45 (0.13-1.14)	0.23 (0.04-0.64)	0.089
CD8+ EM2 PD-1+	7.11 (3.98-10.92)	9.53 (6.09-16.08)	**<0.001**
CD8+ EM2	13.13 (8.19-17.82)	14.86 (10.66-24.760	**0.001**
CD4:CD8	2.18 (1.22-3.49)	3 (1.89-3.70)	0.077

A Wilcoxon signed rank test was performed. All variables are shown as median (interquartile range).

Abbreviations: Cluster of differentiation (CD), effector memory (EM), programmed cell death protein 1 (PD-1).

Differences in the T-cell phenotypes of PLWH between V1 and V2

When running a univariate analysis, PLWH admitted with COVID-19 had higher percentages of CD8+ pre-effector 2 (pE2:CCR7-CD45RA+CD27+CD28+) at V2 (8 ± 1.45 days after admission) when compared to V1 (p-value=0.023). At the V2 timepoint, the percentage of CD8+ EM4 (p-value=0.015) subset, specifically activated CD8+ EM4 (p-value=0.011) T-cells decreased in PLWH. The percentage of activated CD8+ EM3 T-cells decreased, albeit not significantly (p-value=0.050). Supplementary Table S7 and Figure 2 show the differences found between hospital admission and 2.5 ± 3.37 days after admission in PLWH with COVID-19.

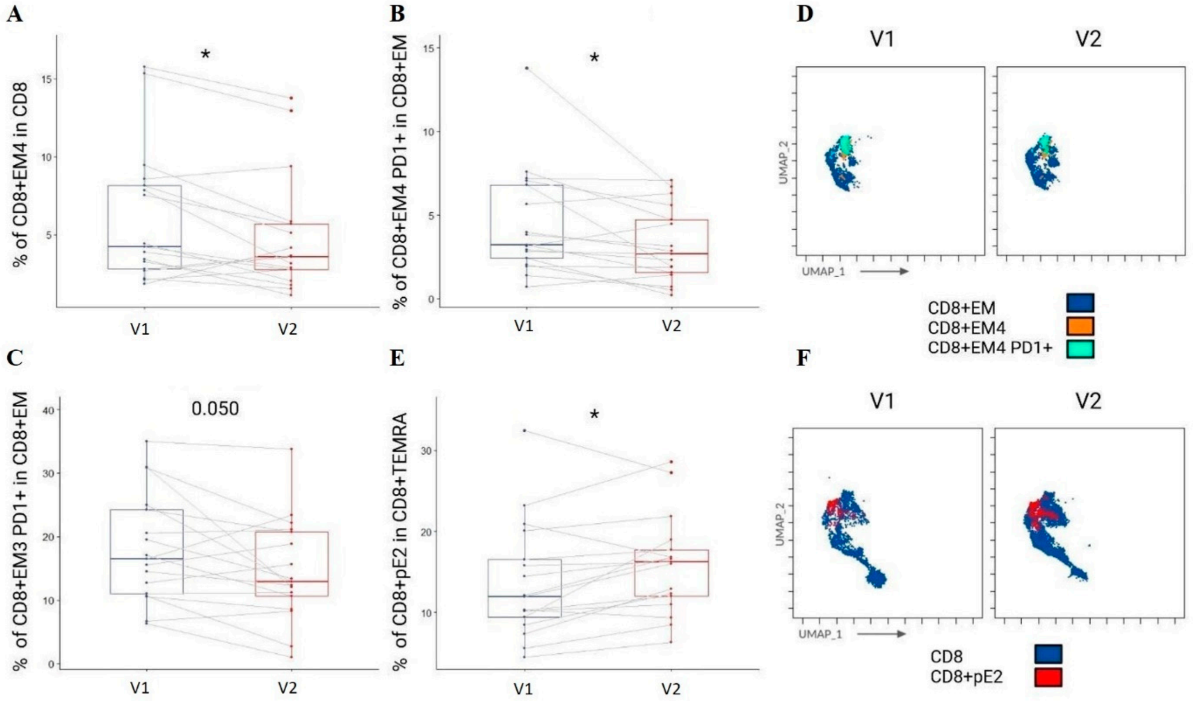

Supplementary Figure S2: Differences in the T-cell phenotypes in PLWH with COVID-19 at hospital admission (V1) and 2.5 ± 3.37 days after admission (V2). **A-C**: Boxplots showing a decrease in the percentages of CD8+ EM4, CD8+ EM4 expressing PD-1 and CD8+ EM3 expressing PD1 at the V2 timepoint. **D**: Representative UMAP plots show the decreases in the respective CD8+ EM T-cell populations between V1 and V2 in PLWH admitted with COVID-19. **E**: The percentage of CD8+ pE2 cells increased from V1 to V2. **F**: Representative UMAP plots show the increase in the CD8+ pre-Effector 2 TEMRA population between V1 and V2 in PLWH admitted with COVID-19.

p-value: * = < 0.05.

Abbreviations: Effector memory (EM), pre-effector (pE), programmed cell death protein 1 (PD1), terminally differentiated T-cells re-expressing CD45RA (TEMRA), visit 1 (V1), visit 2 (V2)

Of the 17 PLWH with a V1 and V2 sample, just under half (8/17- 47.06%) had a CD4+ T-cell count of <200 cells/mm^3 (median 120; IQR: 111-135). There was no significant difference in terms of age (47 ± 14.37 vs 48 ± 8.60, p-value=0.736) and sex (p-value=0.402) between PLWH with a CD4+ T-cell count <200 cells/mm^3 or >200

cells/mm^3. No significant differences were present in terms of co-morbidities in PLWH with a CD4+ T-cell count <200 cells/mm^3: Hypertension (3/8, p-value=0.858), diabetes (2/8, p-value=0.707), cardiovascular disease (1/8, p-value=0.600), pulmonary disease (0/8, p-value=0.331) and tuberculosis (2/8, p-value=0.110). PLWH with a CD4+ T-cell count <200 cells/mm^3 were not hospitalized significantly longer than those patients with a higher CD4+ T-cell count [10 ± 4 days vs 8 ± 5 days, p-value=0.313]. The percentage of CD8+ T-cells was significantly higher at V2 in the group with a lower CD4+ T-cell count (p=0.034). The COVID-19 groups were compared to healthy PLWH with a CD4+ T-cell count of above or below 200 cells/mm^3. Both PLWH COVID-19 groups had significantly higher percentages of activated CD4+ (CD4+ T-cell count <200 cells/mm^3 vs control p-value=0.008, CD4+ T-cell count >200 cells/mm^3 vs control p-value=0.002) and CD8+ (CD4+ T-cell count <200 cells/mm^3 vs control p-value=0.008, CD4+ T-cell count >200 cells/mm^3 vs control p-value<0.001) EM1 subsets when compared to controls. Supplementary Table S7 shows the comparison of the COVID-19 groups with each other.

Next, we analyzed PLWH with a detectable and undetectable viral load (VL). Ten (10/17-58.82%) PLWH admitted with COVID-19 had an undetectable VL (<20 copies/mL) and seven (7/10-41.18%) had a detectable VL. PLWH with a detectable VL were younger (37 ± 12 vs 52 ± 8 years of age, p-value=0.051). In terms of co-morbidities, patients with a detectable VL had significantly lower incidences of diabetes (0/7 vs 5/10, p-value=0.026) and higher incidences of heart disease (4/7 vs 0/10, p-value=0.023). At V2, PLWH with a detectable VL had higher percentages of CD8+ T-cells (p-value=0.023) and lower percentages of CD4+ T-cells (p-value=0.023). Of the CD8+ T-cell subsets, PLWH with detectable VL had significantly lower CD8+ naïve (p-value=0.015), TEMRA (p-value=0.004) and CD8+ EM1 (p-value=0.039) subsets. This group also had significantly higher percentages of activated CD8+ EM3 T-cells (p-value=0.005), specifically CD8+ EM3 T-cells co expressing PD-1 and CD57 (p-value=0.044).

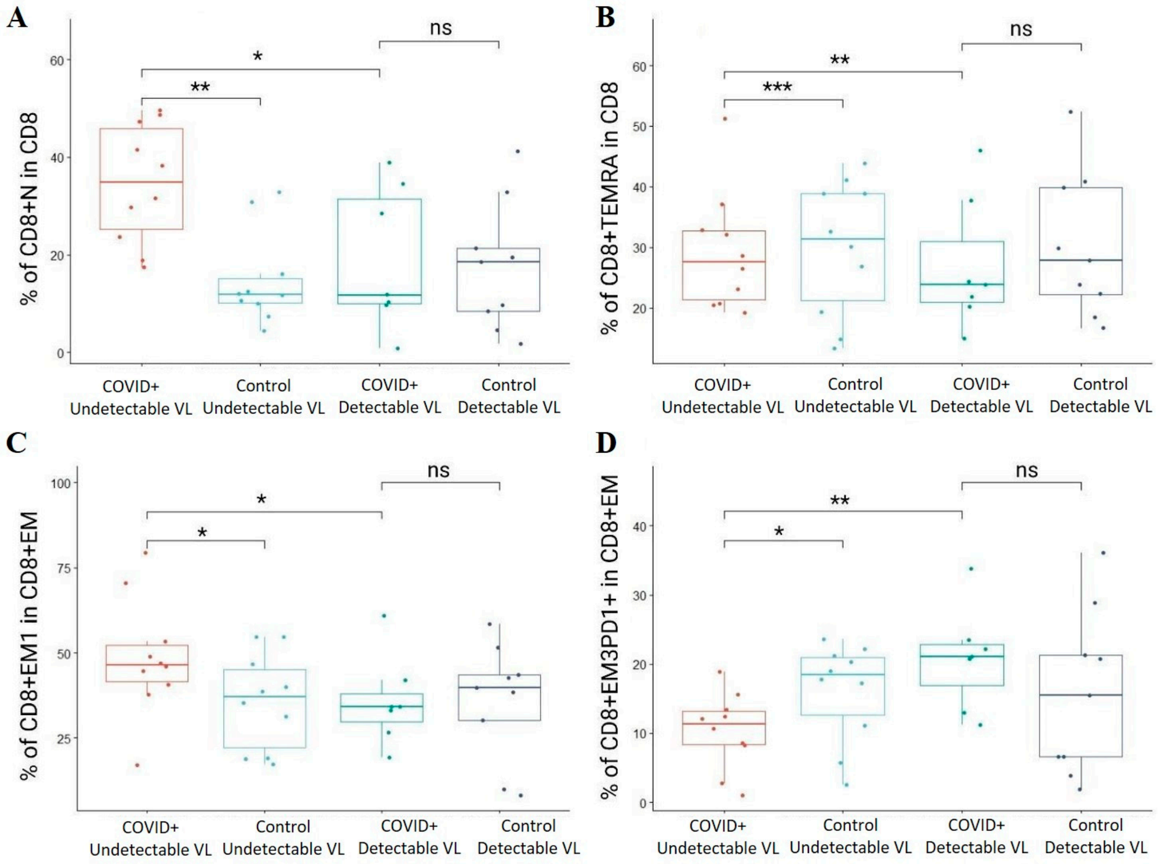

Supplementary Figure S3: Boxplots depicting the differences between PLWH admitted to hospital with COVID-19 with a detectable and undetectable HIV VL, as well as with their respective SARS-CoV-2 negative controls at V2. **A**: PLWH with a detectable VL admitted with COVID-19 had significantly lower CD8+ naïve cells when compared with those that have an undetectable VL. PLWH with COVID-19 had a higher percentage of CD8+ naïve T-cells when compared to PLWH with an undetectable VL without COVID-19. **B**: PLWH with a detectable

VL had lower percentages of CD8+ TEMRA cells when compared to PLWH with an undetectable VL. PLWH with COVID-19 with an undetectable VL had lower percentages of CD8+ TEMRA T-cells when compared to PLWH with an undetectable VL without COVID-19. **C**: PLWH with a detectable VL had lower percentages of CD8+ EM1 T-cells than those with a detectable VL at V2. PLWH with COVID-19 with an undetectable VL had higher percentages of CD8+ EM1 T-cells when compared to PLWH with an undetectable VL without COVID-19. **D**: PLWH with a detectable VL had higher percentages of CD8+EM3 T-cells expressing PD-1 than those with an undetectable VL at V2. PLWH with COVID-19 with an undetectable VL had lower percentages of CD8+ EM3 T-cells expressing PD-1 when compared to PLWH with an undetectable VL without COVID-19.

p-value: * = < 0.05, ** = <0.01, *** = <0.001.

Abbreviations: Effector memory (EM), naïve (N), programmed cell death protein 1 (PD-1), terminally differentiated T-cells re-expressing CD45RA (TEMRA), viral load (VL)

When comparing the VL for each COVID-19 group with their respective controls without COVID-19, PLWH admitted with COVID-19 had lower percentages of CD4+ CM PD-1+ (COVID-19 + detectable VL vs detectable VL control p-value<0.001, COVID-19 + undetectable VL vs undetectable VL control p-value=0.002), CD4+ EM4 (COVID-19 + detectable VL vs detectable VL control p-value=0.009, COVID-19 + undetectable VL vs undetectable VL control p-value=0.002), CD4+ EM4 PD-1+ (COVID-19 + detectable VL vs detectable VL control p-value<0.001, COVID-19 + undetectable VL vs undetectable VL control p-value=0.002), CD4+ TEMRA (COVID-19 + detectable VL vs detectable VL control p-value=0.030, COVID-19 + undetectable VL vs undetectable VL control p-value=0.022) CD8+ N PD-1+ (COVID-19 + detectable VL vs detectable VL control p-value=0.020, COVID-19 + undetectable VL vs undetectable VL control p-value=0.016), CD8+ CM (COVID-19 + detectable VL vs detectable VL control p-value=0.024, COVID-19 + undetectable VL vs undetectable VL control p-value=0.034) T-cells. On the other hand, PLWH admitted with COVID-19 had significantly higher activated CD8+ EM1 (COVID-19 + detectable VL vs detectable VL control p-value=0.008, COVID-19 + undetectable VL vs undetectable VL control p-value<0.001) and CD8+ EM2 CD57+ T-cells.

Supplementary Table S7: Differences in the T-cell phenotypes between V1 and V2 for PLWH admitted to hospital with COVID-19

T-cell population	COVID+ PLWH V1 (n=17)	COVID+ PLWH V2 (n=17)	p-value
CD8+ EM	24.48 (16.43-35.95)	28.04 (25.72-40.84)	0.050
CD8+ E	77.98 (72.48-86.22)	76.61 (68.75-83.64)	0.093
CD8+ pE2	11.21 (9.43-16.51)	16.12 (10.97-17.72)	**0.023**
CD8+ EM4 PD1+	3.19 (2.41-6.81)	2.78 (1.58-4.71)	**0.011**
CD8+ EM3 PD1+	16 (10.68-24.22)	12.65 (8.53-20.77)	0.050
CD8+ EM2	15.79 (11.79-20.51)	16.83 (12.89-25.38)	0.050
CD8+ EM4	4.04 (2.79-8.15)	3.61 (2.76-5.66)	**0.015**

A Wilcoxon signed rank test was performed. All variables are shown as median (interquartile range).

Abbreviations: Effector memory (EM), pre-effector (pE), programmed cell death protein 1 (PD1).

Supplementary Table S8: Comparisons of each COVID-19 group between controls, as well as between PLWH admitted with COVID-19 with a CD4 count of above and below 200 cells/mm³ at the V2 timepoint after performing a Kruskal-Wallis test with post hoc Dunn's test.

T-cell population	CD4 >200 (n=9) Group 1	Control CD4 >200 (n=11) Group 2	CD4<200 (n=8) Group 3	Control CD4<200 (n=7) Group 4	1 vs 2	3 vs 4	1 vs 3	2 vs 4	p-value
DN	4.17 (3.15-5.87)	4.37 (2.73-7.44)	3.67 (2.86-4.78)	6.17 (5.41-9.02)	0.360	**0.010**	0.343	**0.010**	0.106
CD4	58.65 (49.47-62.10)	42.56 (26.12-54.93)	44.95 (23.81-53.74)	46.02 (14.85-64.11)	0.053	0.390	0.050	0.390	0.303
CD8	36.79 (31.75-43.60)	53.13 (39.30-64.98)	50.82 (42.11-70.74)	47.65 (28.00-70.52)	0.065	0.272	**0.038**	0.272	**0.030**
CD4+ TEMRA	0.12 (0.02-0.47)	0.55 (0.19-1.51)	0.02 (0-0.84)	0.55 (0.26-1.39)	**0.044**	**0.024**	0.355	**0.024**	0.073
CD4+ EM	11.97 (11.44-18.33)	23.80 (15.70-38.41)	15.40 (13.33-44.35)	13.57 (12.28-26.00)	**0.003**	0.281	0.085	0.281	**0.041**
CD4+ N	32.84 (26.61-36.70)	16.57 (6.34-33.17)	24.28 (9.09-32.69)	31.41 (19.04-40.83)	**0.024**	0.186	0.091	0.186	0.190
CD4+ CM PD1+	33.19 (25.75-39.73)	48.53 (46.08-74.17)	31.86 (29.26-35.85)	53.01 (46.08-74.18)	**0.006**	**0.006**	0.409	**0.006**	**0.005**
CD4+ EM4	13.08 (7.27-22.02)	28.75 (20.58-35.16)	12.23 (6.59-16.67)	38.73 (30.79-49.61)	**0.014**	**<0.001**	0.347	**0.001**	**0.002**
CD4+ EM1 PD1+	45.27 (29.43-48.86)	10.02 (7.00-34.37)	41.78 (29.43-53.96)	23.47 (8.31-26.39)	**0.002**	**0.008**	0.456	**0.008**	**0.002**
CD4+ EM4 PD1+	5.75 (2.68-8.44)	14.00 (9.79-17.21)	4.50 (3.84-5.70)	26.39 (17.65-30.09)	**0.008**	**<0.001**	0.288	**<0.001**	**<0.001**
CD8+ N	34.49 (17.44-41.61)	12.07 (9.73-18.61)	26.05 (14.26-33.97)	19.50 (8.38-32.89)	**0.006**	0.299	0.160	0.299	0.085
CD8+ TEMRA	23.83 (21.86-32.10)	38.86 (29.89-40.91)	26.49 (19.89-39.41)	18.52 (14.83-27.80)	**0.042**	0.098	0.362	0.097	0.059
CD8+ EM	26.54 (25.72-31.88)	38.44 (32.26-43.60)	37.38 (26.36-44.28)	38.74 (25.47-56.08)	**0.021**	0.322	0.105	0.322	0.190
CD8+ CM	7.24	10.83	5.15	13.84	0.122	**0.007**	0.098	**0.007**	**0.039**

	(5.04-14.86)	(8.08-12.69)	(3.31-8.75)	(5.90-17.05)					
CD8 N PD1+	7.00<br>(4.67-15.16)	20.07<br>(7.60-25.39)	5.33<br>(2.70-8.98)	17.77<br>(10.48-30.58)	**0.034**	**0.010**	0.320	**0.010**	**0.031**
CD8+ pE1	9.03<br>(6.01-16.58)	4.49<br>(2.44-9.32)	4.62<br>(2.95-9.24)	5.55<br>(3.27-13.12)	**0.045**	0.406	0.064	0.406	0.318
CD8+ pE1 PD1+	7.62<br>(2.59-9.71)	2.46<br>(1.44-8.71)	2.65<br>(1.74-6.95)	3.03<br>(2.74-9.83)	**0.048**	0.236	0.086	0.236	0.344
CD8+ E CD57+ PD1+	9.74<br>(4.75-14.12)	12.51<br>(6.07-18.63)	5.93<br>(4.61-7.39)	17.40<br>(7.11-24.86)	0.297	**0.011**	0.152	**0.011**	0.135
CD8+ EM2 CD57+	5.95<br>(4.02-10.89)	2.50<br>(2.26-5.80)	5.87<br>(4.70-6.77)	1.93<br>(1.26-5.81)	**0.032**	**0.043**	0.313	**0..043**	0.081
CD8+ EM3 CD57+ PD1+	9.82<br>(5.81-15.74)	8.03<br>(4.75-14.08)	7.36<br>(5.21-10.13)	16.31<br>(3.50-22.65)	0.333	0.072	0.261	0.072	0.474
CD8+ EM4 CD57+ PD1+	0.99<br>(0.23-2.34)	0.60<br>(0.25-1.21)	0.39<br>(0.22-0.74)	1.81<br>(0.65-3.09)	0.285	**0.008**	0.109	**0.008**	0.098
CD8+ EM4 PD1+	2.70<br>(1.58-4.71)	2.15<br>(1.24-3.44)	2.40<br>(1.20-4.36)	3.19<br>(2.18-8.34)	0.180	0.089	0.267	0.089	0.362
CD8+ EM3 PD1+	18.87<br>(13.37-21.10)	17.82<br>(6.57-21.28)	11.61<br>(9.56-12.65)	20.79<br>(5.73-28.81)	0.420	0.098	0.152	0.098	0.599
CD8+ EM1 PD1+	35.22<br>(29.75-41.05)	1.44<br>(0.84-2.55)	34.98<br>(26.51-45.80)	2.89<br>(1.40-10.64)	**<0.001**	**0.003**	0.452	**0.003**	**<0.001**
CD8+ EM1	44.65<br>(34.21-48.89)	31.38<br>(18.82-40.09)	39.18<br>(30.34-53.43)	42.76<br>(38.40-54.63)	**0.031**	0.396	0.279	0.396	0.259
CD8+ EM4	4.13<br>(2.77-9.39)	5.97<br>(3.20-7.83)	3.35<br>(2.25-4.38)	7.34<br>(3.42-15.96)	0.327	**0.029**	0.138	**0.029**	0.255
CD8+ CM PD1+	61.82<br>(51.76-63.76)	52.27<br>(43.45-65.50)	55.83<br>(46.05-62.46)	59.43<br>(51.97-66.54)	0.199	0.263	0.356	0.263	0.707

The Kruskal-Wallis test with post hoc Dunn's test was used to compare continuous variables between groups. All variables are shown as median (interquartile range) Abbreviation: Central memory (CM), double-positive (DP), end-stage effector (E), effector memory (EM), naïve (N), pre-effector (pE), programmed cell death protein 1 (PD1), terminally differentiated T-cells re-expressing CD45RA (TEMRA).

Supplementary Table S9: Comparison of the COVID-19 groups by VL with each other, as well as their respective controls, at the V2 timepoint after performing a Kruskal-Wallis test with post hoc Dunn test.

T-cell population	Undetectable VL COVID (n=10) Group 1	Undetectable VL Control (n=10) Group 2	Detectable VL COVID(n=7) Group 3	Detectable VL Control (n=9) Group 4	1 vs 2	3 vs 4	1 vs 3	2 vs 4	p-value
CD4+	56.80 (49.47-60.30)	47.55 (26.30-64.19)	33.64 (13.08-53.87)	26.12 (12.83-46.02)	0.175	0.329	**0.023**	**0.044**	**0.042**
CD8+	39.47 (35.68-44.62)	48.80 (29.73-70.11)	59.66 (40.13-79.57)	63.88 (47.65-64.98)	0.181	0.475	**0.023**	0.092	0.089
CD4+ TEMRA	0.06 (0-0.74)	0.60 (0.19-1.51)	0.10 (0-0.47)	0.36 (0.29-1.39)	**0.022**	**0.030**	0.490	0.447	0.052
CD4+ EM	13.66 (9.94-15.59)	18.27 (13.96-27.33)	18.74 (11.83-69.97)	26.00 (17.93-31.79)	**0.034**	0.246	0.067	0.266	0.103
CD4+ CM PD1+	29.51 (25.75-33.81)	48.34 (38.90-59.15)	35.02 (29.89-49.80)	73.03 (47.67-74.17)	**0.003**	**0.014**	0.060	0.071	**<0.001**
CD4+EM4	12.00 (6.41-18.73)	30.74 (23.46-36.25)	13.08 (7.27-23.98)	28.75 (21.22-46.86)	**0.002**	**0.009**	0.473	0.453	**0.003**
CD4+ EM1 PD1+	46.99 (31.43-56.19)	9.16 (7.00-10.97)	33.33 (25.53-48.86)	24.74 (11.17-34.37)	**<0.001**	0.126	0.174	0.062	**<0.001**
CD4+ EM4 PD1+	4.53 (2.68-7.84)	14.58 (10.63-17.65)	4.55 (3.58-9.18)	20.94 (17.21-27.21)	**0.002**	**<0.001**	0.362	0.159	**<0.001**
CD8+ N	34.91 (23.67-47.36)	11.88 (9.98-16.12)	11.79 (9.69-34.49)	18.61 (8.38-21.35)	**0.002**	0.437	**0.015**	0.359	**0.015**
CD8+ TEMRA	27.56 (20.77-32.56)	31.34 (19.42-38.91)	23.83 (20.22-37.69)	27.80 (22.29-39.82)	**<0.001**	0.221	**0.004**	0.469	**0.007**
CD8+ CM	5.69 (4.25-13.31)	11.28 (8.08-15.19)	5.95 (3.33-11.79)	12.69 (10.69-16.12)	**0.034**	**0.024**	0.377	0.474	0.064
CD8+ N PD1+	6.50 (1.97-11.97)	15.58 (8.90-22.71)	5.83 (4.84-24.74)	18.24 (11.42-30.89)	**0.016**	**0.020**	0.295	0.229	**0.018**
CD8+ E CD57+ PD1+	6.63 (5.60-12.94)	16.40 (12.51-22.40)	5.33 (3.86-9.74)	7.11 (3.71-17.40)	**0.036**	0.324	0.370	0.054	0.160

CD8+ EM2 CD57+	5.43<br>(4.02-6.40)	2.41<br>(2.23-2.86)	6.49<br>(4.83-10.89)	3.80<br>(1.26-5.81)	**0.020**	**0.046**	0.442	0.379	0.068
CD8+ EM3 CD57+ PD1+	7.35<br>(5.02-9.82)	11.37<br>(3.49-16.20)	11.43<br>(5.81-19.37)	8.03<br>(4.75-18.36)	0.149	0.206	**0.044**	0.465	0.392
CD8+ EM4 PD1+	2.29<br>(0.71-3.16)	2.19<br>(1.87-3.19)	4.48<br>(1.94-6.68)	2.81<br>(1.24-7.26)	0.424	0.311	0.064	0.184	0.373
CD8+ EM3 PD1+	11.31<br>(8.27-13.37)	18.39<br>(11.09-21.14)	21.10<br>(12.95-23.43)	15.44<br>(6.55-21.28)	**0.047**	0.091	**0.005**	0.357	0.077
CD8+ EM2 PD1+	10.50<br>(9.35-12.06)	4.39<br>(2.99-22.86)	21.28<br>(12.46-28.06)	19.07<br>(5.67-33.25)	0.305	0.325	0.078	0.065	0.202
CD8+ EM1 PD1+	37.12<br>(34.97-42.26)	1.23<br>(0.84-1.84)	29.75<br>(22.86-37.40)	1.23<br>(0.84-1.84)	**<0.001**	**0.008**	0.219	**0.152**	**<0.001**
CD8+ EM1	46.39<br>(40.58-53.28)	37.00<br>(19.21-46.76)	34.12<br>(26.57-41.97)	39.69<br>(30.15-43.48)	**0.047**	0.363	**0.039**	0.451	0.229

The Kruskal-Wallis test with post hoc Dunn's test was used to compare continuous variables between groups. All variables are shown as median (interquartile range).

Abbreviation: Central memory (CM), double-positive (DP), end-stage effector (E), effector memory (EM), naïve (N), pre-effector (pE), programmed cell death protein 1 (PD1), terminally differentiated T-cells re-expressing CD45RA (TEMRA).

Supplementary Table S10: Visit 2 differences in T-cell profiles between PLWH and those without HIV hospitalized with COVID-19 after performing a Kruskal-Wallis test with post hoc Dunn test.

T-cell population	COVID+PLWOH (n=52) Group 1	Control PLWOH(n=10) Group 2	COVID+PLWH (n=17) Group 3	Control PLWH (n=19) Group 4	1 vs 2	1 vs 3	3 vs 4	2 vs 4	p-value
DN	3.03 (2.06-4.29)	3.70 (2.74-5.39)	3.88 (2.77-5.25)	5.47 (3.66-7.44)	0.163	**0.039**	0.071	0.050	**0.003**
CD4+	69.59 (61.93-75.98)	73.01 (65.88-79.63)	52.72 (34.54-58.65)	42.56 (22.23-54.93)	0.292	**<0.001**	0.248	**<0.001**	**<0.001**
CD8+	24.02 (19.92-32.93)	22.92 (15.18-28.04)	43.11 (36.79-59.66)	53.13 (39.30-70.11)	0.296	**<0.001**	0.352	**<0.001**	**<0.001**
CD4+ TEMRA	0.05 (0.01-0.85)	0.15 (0.06-0.45)	0.068 (0-0.47)	0.55 (0.26-1.51)	0.262	0.341	**0.003**	0.065	**0.013**
CD4+ EM	11.82 (8.19-15.95)	7.76 (5.46-9.35)	14.44 (11.83-20.92)	23.36 (13.96-31.79)	**0.034**	0.0572	**0.050**	**<0.001**	**<0.001**
CD4+ CM	57.33 (50.69-63.76)	53.81 (44.70-58.86)	51.27 (47.25-56.57)	51.96 (44.45-59.22)	0.242	**0.015**	0.318	0.298	0.108
CD4+ CM PD1+	20.26 (15.02-26.38)	33.72 (24.64-37.91)	33.26 (28.11-39.73)	53.01 (45.61-73.03)	**0.020**	**<0.001**	**0.007**	**0.003**	**<0.001**
CD4+ EM PD1+	77.56 (70.79-83.57)	61.07 (50.99-70.59)	80.84 (76.81-85.14)	76.18 (65.12-82.58)	**<0.001**	0.114	0.054	**0.008**	**0.002**
CD4+ EM4	9.84 (7.13-14.04)	27.50 (26.18-33.72)	11.54 (6.78-18.73)	30.69 (21.22-38.94)	**<0.001**	0.200	**<0.001**	0.442	**<0.001**
CD4+ EM1	56.29 (43.49-68.43)	67.06 (47.22-73.00)	56.24 (38.82-65.97)	45.69 (20.73-54.26)	0.188	0.247	0.071	**0.006**	**0.035**
CD4+ EM3	19.49 (12.40-36.19)	2.25 (0.30-16.60)	18.26 (13.13-30.72)	16.79 (4.72-42.73)	**0.002**	0.348	0.368	**0.021**	**0.033**
CD4+ EM1 PD1+	41.62 (32.39-51.87)	23.75 (14.49-26.19)	44.50 (29.43-53.11)	10.97 (8.12-29.97)	**<0.001**	0.493	**<0.001**	0.397	**<0.001**
CD4+ EM4 PD1+	4.10 (2.16-5.55)	16.00 (12.97-20.17)	4.50 (3.41-7.84)	17.21 (10.63-26.39)	**<0.001**	0.147	**<0.001**	0.466	**<0.001**
CD8+ N	21.30	20.17	29.06	12.07	0.361	0.154	**0.007**	**0.045**	0.076

	(11.65-35.00)	(15.61-44.65)	(11.79-38.89)	(8.38-21.35)						
CD8+ EM	24.46 (16.12-36.67)	25.98 (14.65-34.88)	28.04 (25.72-40.84)	38.74 (30.90-54.10)	0.406	0.122	**0.045**	**0.006**	**0.007**
CD8+ CM	8.07 (5.68-11.77)	13.39 (7.47-20.88)	5.83 (4.25-11.79)	11.74 (8.08-16.12)	**0.013**	0.147	**0.003**	0.346	**0.006**
CD8+ N PD1+	6.40 (2.86-12.43)	8.41 (3.84-11.43)	6.33 (2.57-9.00)	18.24 (9.32-30.58)	0.380	0.458	**0.001**	**0.008**	**0.001**
CD8+ E	70.60 (51.40-84.19)	47.69 (25.93-77.88)	76.61 (68.75-83.64)	69.63 (56.62-79.34)	0.061	0.193	0.168	0.122	0.273
CD8+ pE2	17.59 (10.04-26.09)	28.34 (16.15-34.67)	16.12 (10.97-17.72)	19.13 (11.34-33.11)	0.066	0.255	0.069	0.296	0.213
CD8+ E CD57+	54.31 (35.04-68.20)	40.29 (21.49-53.80)	48.58 (40.19-60.90)	50.95 (31.87-61.15)	**0.045**	0.311	0.377	0.191	0.364
CD8+ E CD57+ PD1+	7.97 (3.09-11.55)	9.58 (4.29-12.53)	6.40 (4.75-9.74)	14.11 (6.07-21.26)	0.272	0.463	0.066	0.207	0.245
CD8+ EM2 CD57+	7.68 (4.87-11.77)	3.63 (2.23-5.25)	6.14 (4.49-7.42)	2.41 (1.93-5.80)	**0.001**	0.094	**0.018**	0.464	**<0.001**
CD8+ EM2 CD57+ PD1+	4.47 (2.37-7.57)	2.48 (1.73-4.86)	3.90 (2.60-5.46)	3.28 (1.63-5.35)	**0.029**	0.258	0.243	0.271	0.174
CD8+ EM3 CD57+ PD1+	4.63 (1.89-13.91)	4.35 (1.85-9.49)	7.36 (5.02-11.23)	9.30 (3.49-16.31)	0.381	0.084	0.479	0.097	0.265
CD8+ EM4 CD57+ PD1+	0.23 (0.04-0.64)	0.41 (0.14-0.46)	0.46 (0.23-1.38)	0.65 (0.34-1.81)	0.450	**0.016**	0.221	**0.019**	**0.006**
CD8+ EM4 PD1+	1.26 (0.58-2.10)	4.15 (3.25-6.38)	2.78 (1.58-4.71)	2.23 (1.24-3.73)	**<0.001**	**0.003**	0.460	**0.021**	**<0.001**
CD8+ EM3 PD1+	6.83 (3.42-16.97)	5.55 (2.66-11.13)	12.65 (8.53-20.77)	17.82 (6.55-21.28)	0.221	**0.033**	0.446	**0.017**	**0.040**
CD8+ EM2 PD1+	9.53 (6.09-16.08)	6.72 (4.51-9.93)	11.86 (9.35-21.28)	11.00 (3.41-33.25)	0.064	0.072	0.145	0.069	0.136
CD8+ EM1 PD1+	39.03 (29.08-50.17)	3.44 (2.64-5.13)	35.12 (29.75-42.26)	1.64 (0.86-3.29)	**<0.001**	0.272	**<0.001**	0.218	**<0.001**

CD8+ EM1	49.51 (38.25-64.36)	64.74 (59.49-70.49)	43.31 (34.12-53.28)	38.64 (19.21-46.76)	0.064	0.067	0.136	**<0.001**	**0.002**
CD8+ EM2	14.86 (10.66-24.760	11.55 (7.28-14.80)	16.83 (12.89-25.38)	18.13 (8.69-43.22)	0.051	0.152	0.488	**0.014**	0.110
CD8+ EM4	4.08 (2.73-5.21)	7.68 (5.68-9.35)	3.61 (2.76-5.66)	5.97 (3.20-9.00)	**0.001**	0.373	0.075	**0.041**	**0.009**
CD8+ EM3	21.18 (8.69-40.23)	12.08 (5.38-23.72)	28.95 (22.64-39.28)	27.70 (12.99-47.45)	0.098	0.073	0.382	**0.027**	0.118
CD8+ CM PD1+	45.65 (38.63-54.42)	51.26 (42.11-54.99)	58.60 (46.87-65.42)	55.34 (44.71-65.50)	0.326	**0.005**	0.398	0.114	**0.022**

The Kruskal-Wallis test with post hoc Dunn's test was used to compare continuous variables between groups. All variables are shown as median (interquartile range)

Abbreviation: Central memory (CM), double-positive (DP), end-stage effector (E), effector memory (EM), naïve (N), pre-effector (pE), programmed cell death protein 1 (PD1), terminally differentiated T-cells re-expressing CD45RA (TEMRA).

S2.1 Extra flow cytometry information

S2.1.1 Panel design: Fluorescent antibodies used and compensation

Supplementary Table S11: Fluorescent reagents used in the T-cell panel.

Characteristic	Analyte	Detector	Reporter	Manufact.	Clone	Cat#
T helper	CD4	Anti-CD4	APC	Beckman Coulter	13B8.2	B53328
T cytotoxic	CD8	Anti-CD8	AF-700	Beckman Coulter	B9.11	B53328
T lineage marker	CD3	Anti-CD3	APC-750	Beckman Coulter	UCHT-1	B53328
Common leukocyte antigen	CD45RO	Anti-CD45RO	Krome Orange	Beckman Coulter	J33	B53328
Common leukocyte antigen	CD45RA	Anti-CD45RA	FITC	Beckman Coulter	2H4	B53328
T-cell homing marker	CD197 (CCR7)	Anti-CD197	PE	Beckman Coulter	G043H7	B53328
Co-stimulatory ligand to CD80 and CD86	CD28	Anti-CD28	ECD	Beckman Coulter	CD28.2	B53328
Co-stimulatory molecule	CD27	Anti-CD27	PC7	Beckman Coulter	1A4.CD27	B53328
T-cell exhaustion marker	CD57	Anti-CD57	Pacific Blue	Beckman Coulter	NC1	B53328
T-cell activation marker	CD279 (PD-1)	Anti-CD279	PC5.5	Beckman Coulter	PD1.3.5	B53328

Abbreviations: Alexa fluor (AF), Allophycocyanin (APC), C-C chemokine receptor type 7 (CCR7), cluster of differentiation (CD), Phycoerythrin-Texas red conjugate (ECD), Fluorescein isothiocyanate (FITC), fluorescence channel (FL), Phycoerythrin cyanine (PC), Programmed cell death protein 1 (PD-1), Phycoerythrin (PE).

Compensation set up (Supplementary Table S12) was performed by utilizing the compensation kit provided as part of the Duraclone kit by staining whole blood according to the manufacturer's instructions. bi-exponential plots of scaling and compensation can be seen in Supplementary Figure S4.

Supplementary Table S12: Compensation tubes have been created as follows.

Optical detector	*FL9*	*FL10*	*FL1*	*FL2*	*FL3*	*FL4*	*FL5*	*FL6*	*FL7*	*FL8*
Reporter	*Pacific Blue*	*Krome Orange*	*FITC*	*PE*	*ECD*	*PC5.5*	*PC7*	*APC*	*AF-700*	*AF750*
Tube #1	+Ab	-	-	-	-	-	-	-	-	-
Tube #2	-	+Ab	-	-	-	-	-	-	-	-
Tube #3	-	-	+Ab	-	-	-	-	-	-	-
Tube #4	-	-	-	+Ab	-	-	-	-	-	-
Tube #5	-	-	-	-	+Ab	-	-	-	-	-
Tube #6	-	-	-	-	-	+Ab	-	-	-	-
Tube #7	-	-	-	-	-	-	+Ab	-	-	-
Tube #8	-	-	-	-	-	-	-	+Ab	-	-
Tube #9	-	-	-	-	-	-	-		+Ab	-
Tube #10	-	-	-	-	-	-	-	-		+Ab

Abbreviations: antibody (Ab), Alexa fluor (AF), Allophycocyanin (APC), Phycoerythrin-Texas red conjugate (ECD), Fluorescein isothiocyanate (FITC), fluorescence channel (FL), Phycoerythrin cyanine (PC), Phycoerythrin (PE).

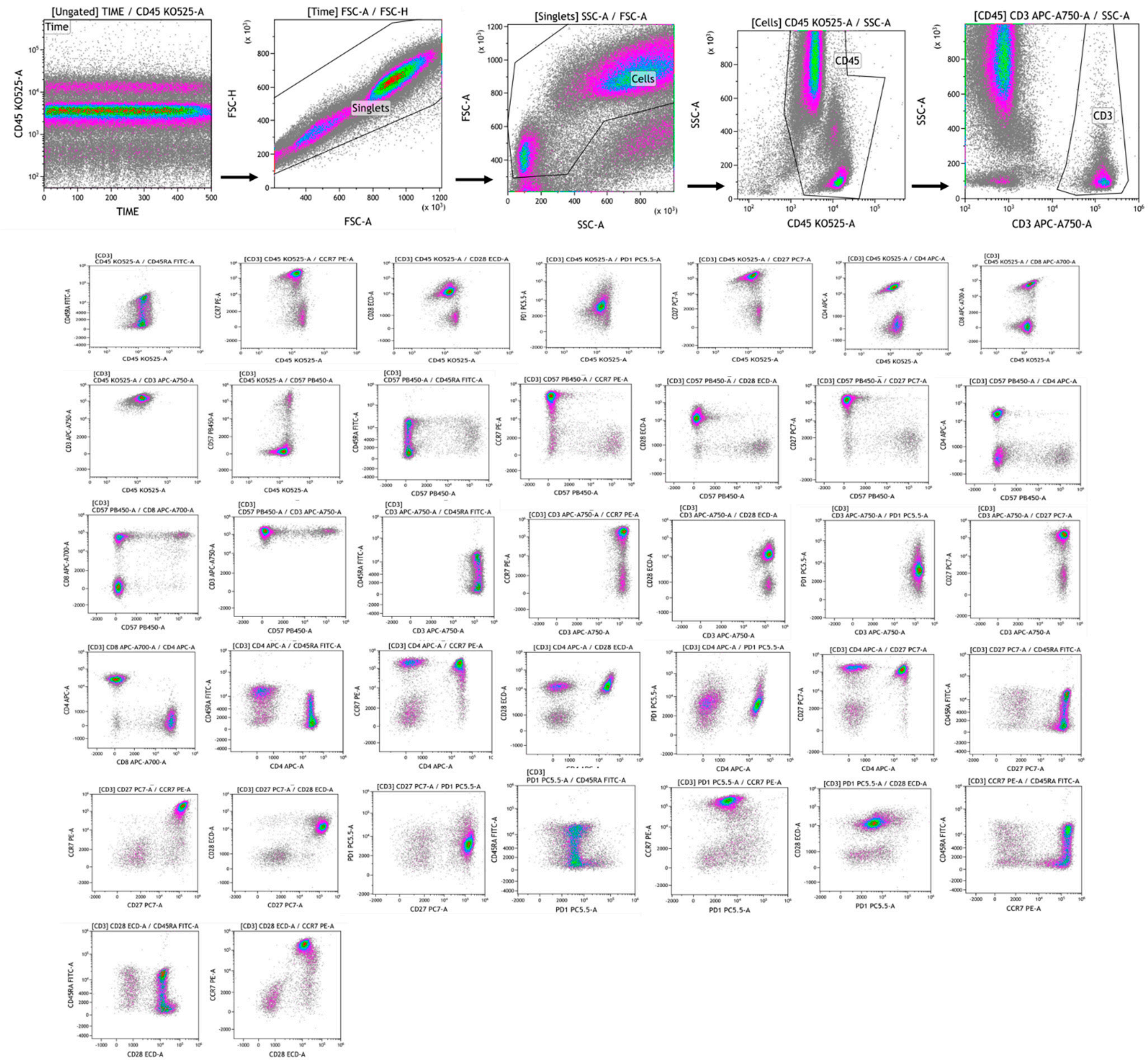

Supplementary Figure S4: Example of data clean-up gating strategy and compensation for T-cells. Each axis was changed to logicle to ensure that all of the data can be seen on the plot. Each marker was plotted against one another to visualize the compensation before uploading to Cytobank.

S2.1.2 Cytobank analysis

S2.1.2.1 PeacoQC was used on all the FCS files uploaded to Cytobank

After uploading the files onto the Cytobank platform an additional quality check was performed by running the PeacoQC algorithm. PeacoQC identifies and removes any irregularities such asupregulated or downregulated signal during the acquisition. of the data and creates a new file which it labels as a “Cleaned file”.

S2.1.2.2 UMAP was used to visualize and gate the data in Cytobank

On the “Cleaned files” generated by the PeacoQC algorithm, the lymphocyte population was generated by plotting SSC vs CD45RO (Supplementary Figure S6A). Next by plotting SSC vs CD3, the T-cell population was gated by gating on the CD3+ population. The CD3+ population was selected as the input parameter for the UMAP algorithm. UMAP settings included:

- Channels to be included: CCR7-PE, CD27-PC7, CD28-ECD, CD4-APC, CD8 APC-A700, CD45RA, CD57-Pacific Blue and PD1-PC5.5

- Events to be sampled: All events in CD3 gate (total of 3 580 511 across all files)
- Advanced settings included: Number of neighbors:20, minimum distance between clusters:0,01 and we selected to collapse outliers
- The data was normalized and the internal file compensation was applied

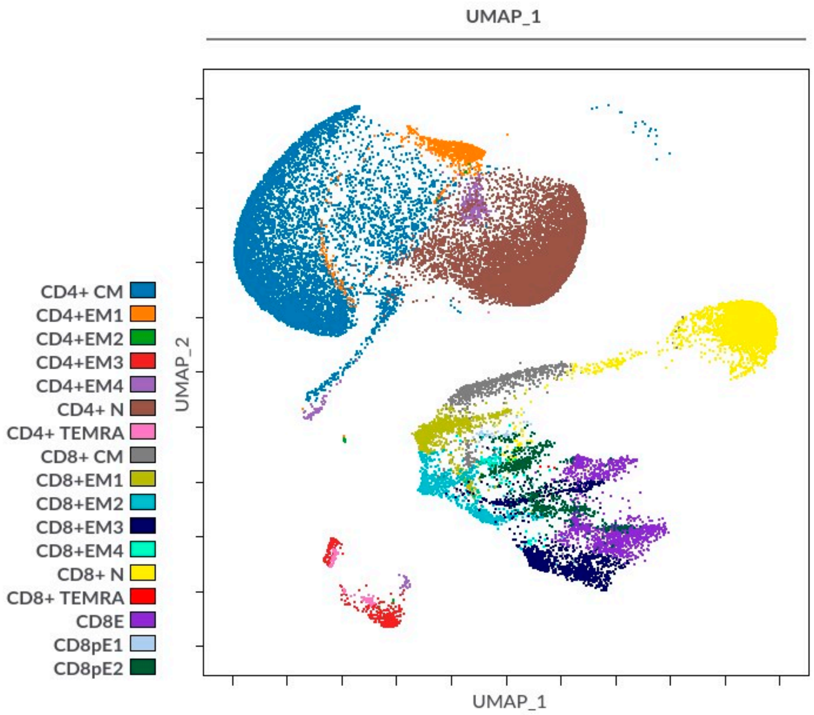

Supplementary Figure S5: Example of UMAP output generated of T-cell populations identified.

S2.1.2.3 CITRUS

CITRUS was used to identify significant biological differences between PLWH and those without HIV admitted with COVID-19.

After performing the UMAP analysis CITRUS was run as downstream analysis. Two CITRUS experiments were setup, one in which the input gate was CD4 and one in which the input gate was CD8. CITRUS performs statistical analysis thus the groups and files to be compared need to be specified. PLWH and people without HIV admitted with COVID-19 were matched for sex and age within STATA 17 and the exact matched files were selected to be compared with one another in the CITRUS experiment. Thus 36 files from both groups were selected (PLWH and people without HIV) and labelled "HIV+" and "HIV-". Clustering channels chosen include: CCR7-PE, CD27-PC7, CD28-ECD, CD45RA, CD57-Pacific Blue and PD1-PC5.5. Internal file compensation was used (refer to above mentioned compensation matrix Supplementary Figure S4). Association model specified: Significance Analysis of Microarrays (SAM) – Correlative. Cluster characterization was set to abundance. Equal event sampling was selected.

S2.1.2.4 Gate Description T-cell panel

The gating strategy involves the following gates:
- SSC vs CD45KO- lymphocyte gate to define the lymphocytes (Supplementary Figure S6A).
- SSC vs CD3- leukocyte gate applied- gate CD3 to define the T-cells (Supplementary Figure S6B).
- CD8 vs CD4- CD3 gate applied- create a quadrant gate to differentiate between CD4, CD8, DP and DN T-cells. (Supplementary Figure S6C).
- CD4 gate applied:
-CCR7 vs CD45RA to define the different CD4 memory subsets: naïve (CD4 N), central memory (CD4 CM), effector memory (CD4 EM) and terminal effector cells re-expressing CD45RA (CD4 TEMRA). (Supplementary Figure S6D).

-CD4 EM gate applied:
-CD28 vs CD27 to define the different effector memory subsets: EM1 (CD4 EM1), EM2 (CD4 EM2), EM3 (CD4 EM3), EM4 (CD4 EM4). (Supplementary Figure S6E)
- CD8 gate applied- CCR7 vs CD45RA to define the different CD8 memory subsets: naïve (CD8 N), central memory (CD8 CM), effector memory (CD8 EM) and terminal effector cells re-expressing CD45RA (CD8 TEMRA). (Supplementary Figure S6F).
-CD8 EM gate applied:
-CD28 vs CD27 to define the different effector memory subsets: EM1 (CD8 EM1), EM2 (CD8 EM2), EM3 (CD8 EM3), EM4 (CD8 EM4). (Supplementary Figure S6G)
-CD8 TEMRA gate applied
-CD28 vs CD27 to define the different TEMRA subsets pre-effector 1 (CD8 pE1), pre-effector 2 (CD8 pE2), end-stage effectors (CD8 E). (Supplementary Figure S6H)
- The following gates were drawn on the UMAP1 vs UMAP2 plot by using the Z-channel coloring: CD4 CM PD1+, CD4 EM1 PD1+, CD4 EM2 PD1+, CD4 EM3 PD1+, CD4 EM4 PD1+, CD4 EM3 PD1+CD57+, CD8 CM PD1+, CD8 EM1 PD1+, CD8 EM2 PD1+, CD8 EM3 PD1+, CD8 EM1 CD57+, CD8 EM2 CD57+, CD8 EM3 CD57+, CD8 EM4 CD57+, CD8 TEMRA pE1 PD1+, CD8 TEMRA E PD1+, CD8 TEMRA E PD1+CD57+. (Supplementary Figure S6).

S2.1.2.5 Gate Boundaries

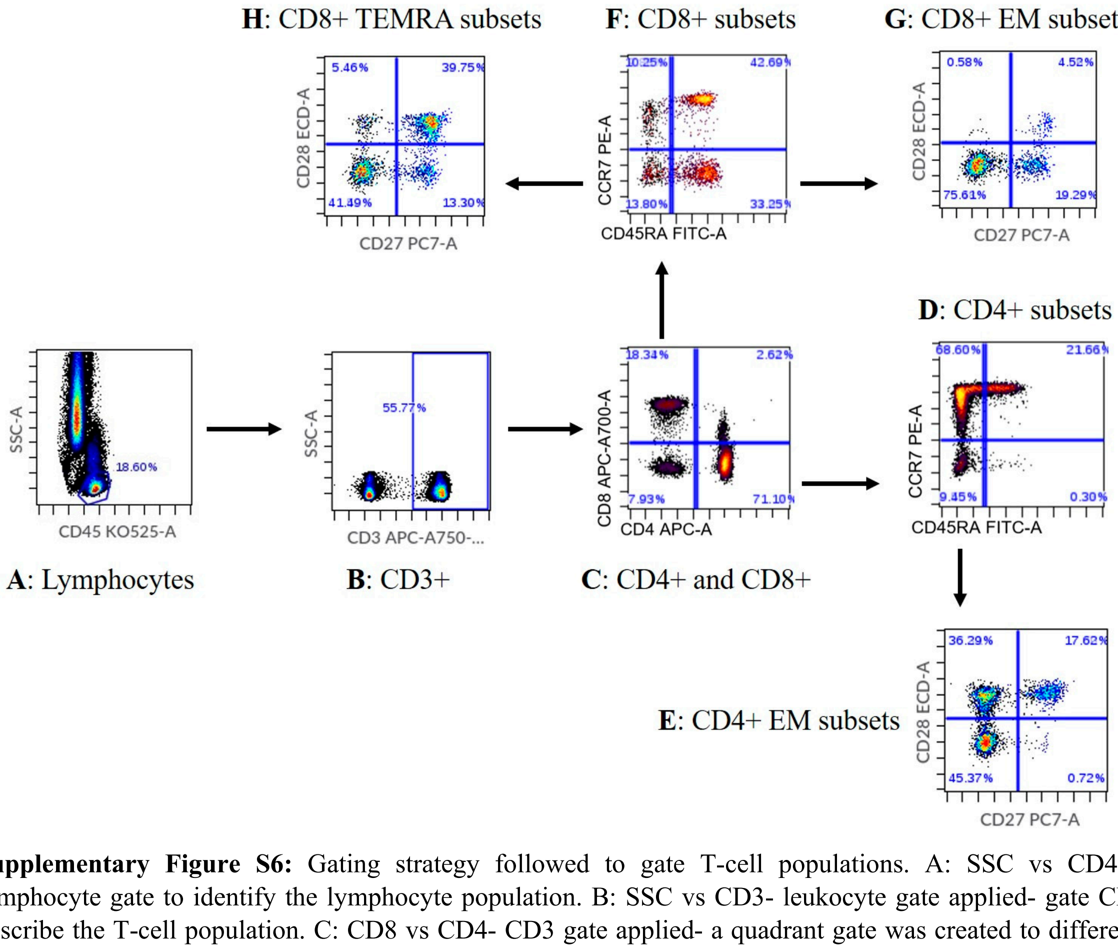

Supplementary Figure S6: Gating strategy followed to gate T-cell populations. A: SSC vs CD45KO- lymphocyte gate to identify the lymphocyte population. B: SSC vs CD3- leukocyte gate applied- gate CD3 to describe the T-cell population. C: CD8 vs CD4- CD3 gate applied- a quadrant gate was created to differentiate between CD4, CD8, DP and DN T-cells. D: CCR7 vs CD45RA to gate the different CD4 memory subsets Naïve (CD4 N), central memory (CD4 CM), effector memory (CD4 EM) and terminal effector cells re-expressing CD45RA (CD4 TEMRA). E: CD28 vs CD27 to define the different effector memory subsets EM1 (CD4 EM1), EM2 (CD4 EM2), EM3 (CD4 EM3), EM4 (CD4 EM4). F: CCR7 vs CD45RA to define the different CD8 memory subsets CD8 N, CM, EM and TEMRA. G: CD28 vs CD27 to define the different effector memory subsets EM1,

EM2 , EM3 , EM4. H: CD28 vs CD27 to define the different TEMRA subsets pre-effector 1 (pE1), pre-effector 2 (pE2), end-stage effectors (E).
